# Heparin promotes fibrillation of most phenol-soluble modulin virulence peptides from *Staphylococcus aureus*

**DOI:** 10.1016/j.jbc.2021.100953

**Published:** 2021-07-14

**Authors:** Zahra Najarzadeh, Masihuz Zaman, Vita Sereikaite, Kristian Strømgaard, Maria Andreasen, Daniel E. Otzen

**Affiliations:** 1 Interdisciplinary Nanoscience Centre (iNANO), Aarhus University, Aarhus C, Denmark; 2 Department of Biomedicine, Aarhus University, Aarhus C, Denmark; 3 Department of Drug Design and Pharmacology, University of Copenhagen, Copenhagen Ø, Denmark

**Keywords:** phenol-soluble modulin, bacterial amyloid, heparin, PSM peptides, biofilm, *Staphylococcus aureus*, ATR, attenuated total internal reflection, eDNA, extracellular DNA, PSM, phenol-soluble modulin, SRCD, synchrotron radiation circular dichroism, TEM, transmission electron microscopy, ThT, thioflavin T, TSB-T, Tris saline buffer with 0.1% Tween-20

## Abstract

Phenol-soluble modulins (PSMs), such as α-PSMs, β-PSMs, and δ-toxin, are virulence peptides secreted by different *Staphylococcus aureus* strains. PSMs are able to form amyloid fibrils, which may strengthen the biofilm matrix that promotes bacterial colonization of and extended growth on surfaces (*e.g.*, cell tissue) and increases antibiotic resistance. Many components contribute to biofilm formation, including the human-produced highly sulfated glycosaminoglycan heparin. Although heparin promotes *S. aureus* infection, the molecular basis for this is unclear. Given that heparin is known to induce fibrillation of a wide range of proteins, we hypothesized that heparin aids bacterial colonization by promoting PSM fibrillation. Here, we address this hypothesis using a combination of thioflavin T-fluorescence kinetic studies, CD, FTIR, electron microscopy, and peptide microarrays to investigate the mechanism of aggregation, the structure of the fibrils, and identify possible binding regions. We found that heparin accelerates fibrillation of all α-PSMs (except PSMα2) and δ-toxin but inhibits β-PSM fibrillation by blocking nucleation or reducing fibrillation levels. Given that *S. aureus* secretes higher levels of α-PSM than β-PSM peptides, heparin is therefore likely to promote fibrillation overall. Heparin binding is driven by multiple positively charged lysine residues in α-PSMs and δ-toxins, the removal of which strongly reduced binding affinity. Binding of heparin did not affect the structure of the resulting fibrils, that is, the outcome of the aggregation process. Rather, heparin provided a scaffold to catalyze or inhibit fibrillation. Based on our findings, we speculate that heparin may strengthen the bacterial biofilm and therefore enhance colonization *via* increased PSM fibrillation.

Functional bacterial amyloids are proteins secreted from bacteria, which self-assemble to form highly ordered β-sheet–rich fibrils or amyloids. These fibrils promote bacterial biofilm formation by acting as structural scaffolds in the biofilm matrix, leading to increased antibiotic resistance (1). The most well-understood functional bacterial amyloids are curli in *Escherichia coli* (2), Fap in *Pseudomonas* (3), TasA in *Bacillus subtilis* (4), and phenol-soluble modulins (PSMs) in *Staphylococcus* strains (5). Unlike the other amyloids, PSMs are short peptides with multiple functions. Besides their ability to strengthen biofilm through amyloid formation, they act as virulence factors that lyse neutrophils and erythrocytes and stimulate inflammatory responses (6, 7, 8). As amphipathic peptides, they are both surface and membrane active, and this is thought to lead to cell permeabilization, as well as encouraging early steps in biofilm formation (5, 9). PSMs are classified according to their length: the shortest (20–25 residues) and most abundant are the four α-PSMs and δ-toxin, which all adopt an α-helical amphipathic structure in solution. The two β-PSMs (∼44 residues), both containing a C-terminal amphipathic α-helix, are found in much lower amounts *in vivo* (10).

Extracellular fibrils isolated from *Staphylococcus aureus* biofilm contain several different PSMs (5). Almost all individual PSMs fibrillate in the classic cross-β amyloid motif with β-strands perpendicular to the fibril axis. The only exceptions are PSMα2 and δ-toxin, which do not fibrillate on their own, and PSMα3, which is the first reported example of the cross-α fold, in which monomeric α-helices are oriented perpendicularly to the fibril axis (9, 11). Preformed PSM fibrils (particularly those formed by PSMα1) accelerate fibrillation of other PSM peptides and even seed fibrillation of PSMα2 and δ-toxin (11). PSMα3 fibrillates very rapidly and thus provides seeds to promote fibrillation of PSMs such as PSMα1 (despite the difference in structure), which in turn is very efficient at accelerating the fibrillation of other PSMs (11).

Besides inter-PSM interactions, other components found in the biofilm matrix, such as polysaccharides, proteins, and extracellular DNA (eDNA), may influence fibrillation. eDNA is known to promote PSMα1 fibrillation (12); similarly, bacterial biosurfactants such as rhamnolipids and outer-membrane lipopolysaccharides generally promote amyloid formation (13). Importantly, eukaryotic host factors can also play a role. Chief among these is heparin, a glycosaminoglycan that, thanks to its many sulfate and carboxyl groups, is the most highly anionic biomacromolecule known (14). Heparin is normally stored intracellularly in secretory granules and released upon tissue injury to act as an anticoagulant, preventing clot formation by fibrinogen (15, 16). This has inspired its use as an anticoagulant in patient catheters, especially for kidney dialysis. In a bacterial context, however, heparin stimulates *S. aureus* biofilm formation by accumulating in the biofilm matrix, probably by binding to cell-surface proteins as a mimic of eDNA. This often leads to catheter infections (17, 18, 19). *In vitro* heparin effectively induces fibrillation of a range of amyloidogenic proteins, for example, lysozyme, α-synuclein, human islet amyloid polypeptide, human muscle acylphosphatase, transthyretin, β_2_-microglobulin, Aβ, and the prion protein (20, 21, 22, 23, 24, 25, 26). These observations prompted us to hypothesize that heparin might encourage biofilm formation through PSM fibrillation. Here, we investigate how heparin affects PSM fibrillation processes by a combination of biophysical techniques, peptide arrays, and biofilm formation assays.

## Results

### Aggregation kinetics of different PSMs is influenced by heparin

To investigate the effect of heparin on the fibrillation of all seven PSM peptides (PSMα1–4, PSMβ1 and PSMβ 2, and δ-toxin) (Table 1), we incubated all PSMs individually (at fixed monomeric concentration) with different concentrations of heparin under quiescent conditions. Aggregation kinetics were monitored using the amyloid-binding dye thioflavin T (ThT) (27). We have previously reported that PSMα1, PSMα3, PSMβ1, and PSMβ2 reproducibly aggregate to ThT-binding amyloid fibrils in the absence of heparin on the hour scale under quiescent conditions with different nucleating mechanisms (11), and peptide concentrations were chosen in accordance with these studies. In the case of PSMβ1 (which was studied at ca. 10-fold lower concentrations than the other peptides), higher concentrations would lead to a kinetic regime where the aggregation kinetics are independent of the peptide concentration because of saturation effects and therefore not suitable to study the effects of other molecules present during aggregation. The addition of high–molecular-weight heparin dramatically changes the aggregation kinetics of all PSMs, but the effect varies between PSMs.

**Table 1:** Sequences and charges of PSM peptides

Peptides	Sequence	Number of residues	Net charge (pH 7)	Positive residues (Arg + Lys)	Negative residues (Asp + Glu)	pI
PSMα1	MGIIAGIIKVIKSLIEQFTGK	21	+2	3	1	9.7
PSMα2	MGIIAGIIKFIKGLIEKFTGK	21	+3	4	1	10
PSMα3	MEFVAKLFKFFKDLLGKFLGNN	22	+2	4	2	9.5
PSMα4	MAIVGTIIKIIKAIIDIFAK	20	+2	3	1	9.7
δ-toxin	MAQDIISTIG DLVKWIIDTV NKFTKK	26	+1	4	3	8.2
PSMβ1	MEGLFNAIKD TVTAAINNDG AKLGTSIVSI VENGVGLLGK LFGF	44	−1	3	4	4.7
PSMβ2	MTGLAEAIAN TVQAAQQHDS VKLGTSIVDI VANGVGLLGK LFGF	44	−0.9	3	2	5.3

**Figure 1:**
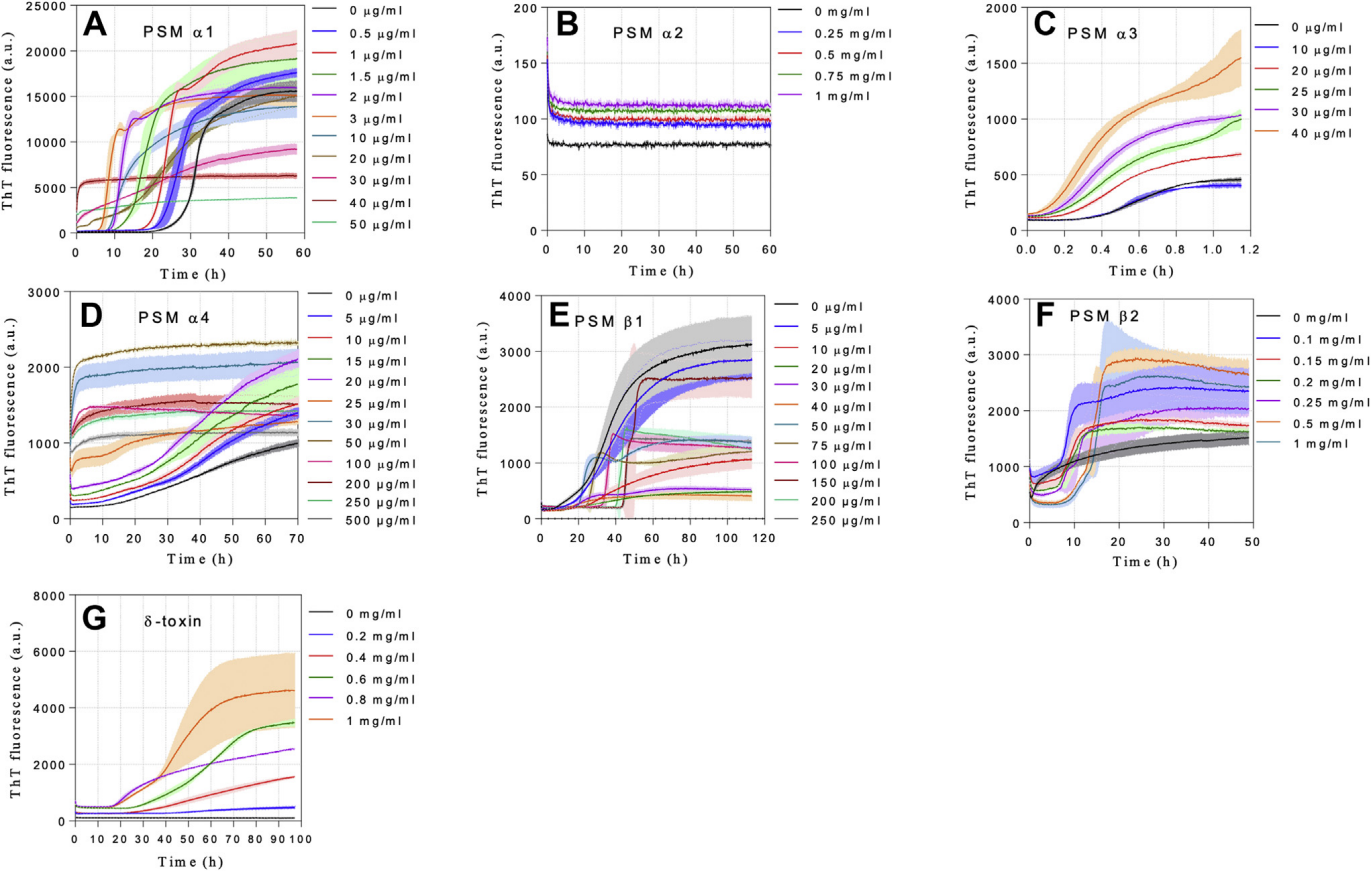
**Thioflavin T time curves for PSM aggregation under quiescent conditions in the absence and presence of different heparin concentrations.** The data depict representatives of triplicate experiments. Heparin concentrations are indicated to the right of each graph (*A–G*). PSMα1–4 and PSMβ2 were incubated at 0.25 mg/ml PSM while PSMβ1 and δ-toxin were incubated at 0.025 and 0.3 mg/ml peptide, respectively. PSM, phenol-soluble modulin.

For PSMα1, as little as 1.0 μg/ml heparin increases end-level ThT fluorescence intensity and decreases the timescale for the completion of aggregation kinetics from ∼40 to ∼25 h (Fig. 1*A*). Heparin also reduces the lag time from 22 h (heparin free) to 7 h (3.0 μg/ml heparin) in a dose-dependent manner (Fig. 1*A*). The lag phase decreases to 1 to 3 h up to 20 μg/ml heparin, above which it is completely abolished, accompanied by a reduction in ThT end-point fluorescence (Fig. 1*A*).

PSMα2 was not observed to undergo fibrillation (measured as an increase in ThT fluorescence) in the absence or the presence of up to 1 mg/ml of heparin (Fig. 1*B*). In contrast, PSMα3 fibrillated readily and with a sigmoidal time curve both in the absence and presence of 0 to 50 μg/ml heparin under quiescent conditions. Heparin significantly reduced lag times and correspondingly increased end-point ThT fluorescence. Thus, 40 μg/ml of heparin induced a fivefold increase in fluorescence and an approximately fourfold reduced lag time (Fig. 1*C*).

The last α-PSM construct (PSMα4) shows the same ThT kinetics up to 20 μg/ml heparin though with an increase in overall ThT fluorescence (Fig. 1*D*). However, when the ThT signals are normalized, the signals collapse to the same time curve. Above 25 μg/ml heparin, the lag time is abolished (Fig. 1*D*), and the data for 30 to 500 μg/ml heparin could be fitted with an exponential decay. The resultant rate constant (*k*) and amplitude of the reaction (A) decreased with [heparin] (Fig. S1).

For PSMβ1, a more complex scenario is revealed. In the absence of heparin, the peptide fibrillates with a lag time of ∼8 h (Fig. 1*E*). This increases to ∼18 h in 10 to 40 μg/ml heparin with a major decrease in ThT fluorescence intensity (Fig. 1*E*). There is then an abrupt shift around 50 μg/ml where the lag time remains ∼20 h but with a much shorter and steeper elongation phase, leading to a medium level of ThT fluorescence. Increasing [heparin] above 250 μg/ml only slightly increases lag times. PSMβ2 fibrillates with a lag time of ∼1 h in the absence of heparin. Heparin increases both the lag time and the end-point ThT fluorescence (Fig. 1*F*). δ-toxin on its own did not show any ThT fluorescence increase, whereas heparin dramatically increased its ThT intensity with lag times decreasing from ∼45 h (0.2–0.4 mg/ml heparin) to ∼18 to 22 h (1 mg/ml heparin) (Fig. 1*G*).

### Fitting of ThT curves using AmyloFit reveals the predominant mechanism of fibrillation kinetics

**Figure 2:**
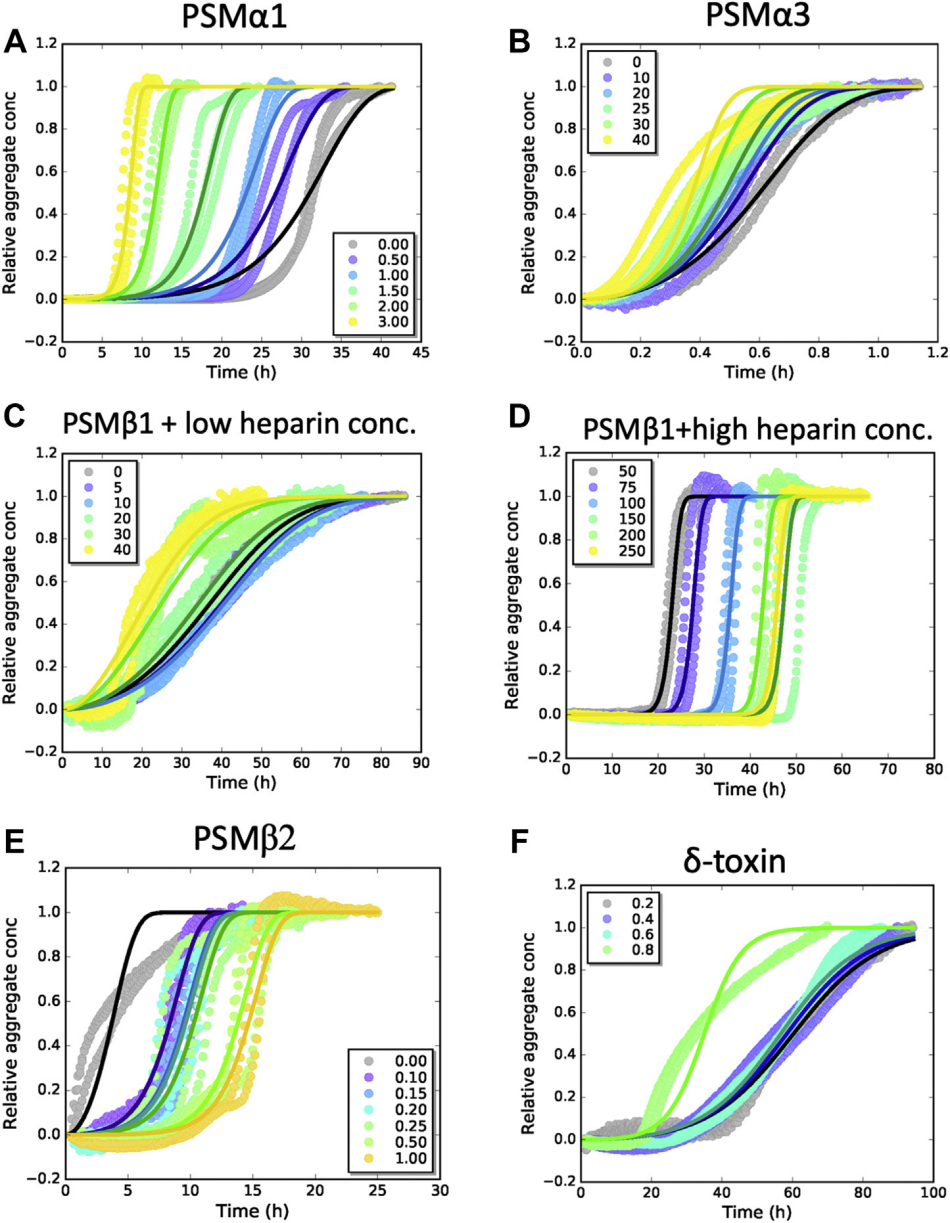
**Fitting of aggregation kinetic data for PSM peptides in the presence of heparin using AmyloFit.** The heparin concentration in microgram/milliliter is indicated for each curve, all data fitted to a secondary nucleation–dominated model using global constants: *n*_c_, *n*_2_, *k*_+_*k*_*n*_, individual fit: k_+_k_2_ (for PSMβ1: individual fit: *k*_+_*k*_*n*_ and global constant *k*_+_*k*_2_). *A*, fitting of PSMα1 kinetic data at 0.25 mg/ml PSMα1 in the presence of 0 to 3 μg/ml heparin. *B*, fitting of PSMα3 kinetic data at 0.25 mg/ml PSMα3 in the presence of 0 to 40 μg/ml heparin. *C*, fitting of PSMβ1 kinetic data at 0.025 mg/ml PSMβ1 in the presence of 0 to 40 μg/ml heparin. *D*, fitting of PSMβ1 kinetic data at 0.025 mg/ml PSMβ1 in the presence of 50 to 250 μg/ml heparin. *E*, fitting of PSMβ2 kinetic data at 0.25 mg/ml PSMβ2 in the presence of 0.1 to 1 μg/ml heparin. *F*, fitting of δ-toxin kinetic data at 0.3 mg/ml δ-toxin in the presence of 0.2 to 0.8 μg/ml heparin fitted to a secondary nucleation–dominated model using global constants: *n*_c_, *n*_2_, *k*_+_*k*_*n*_, individual fit: *k*_+_*k*_2_. PSM, phenol-soluble modulin.

**Figure 3:**
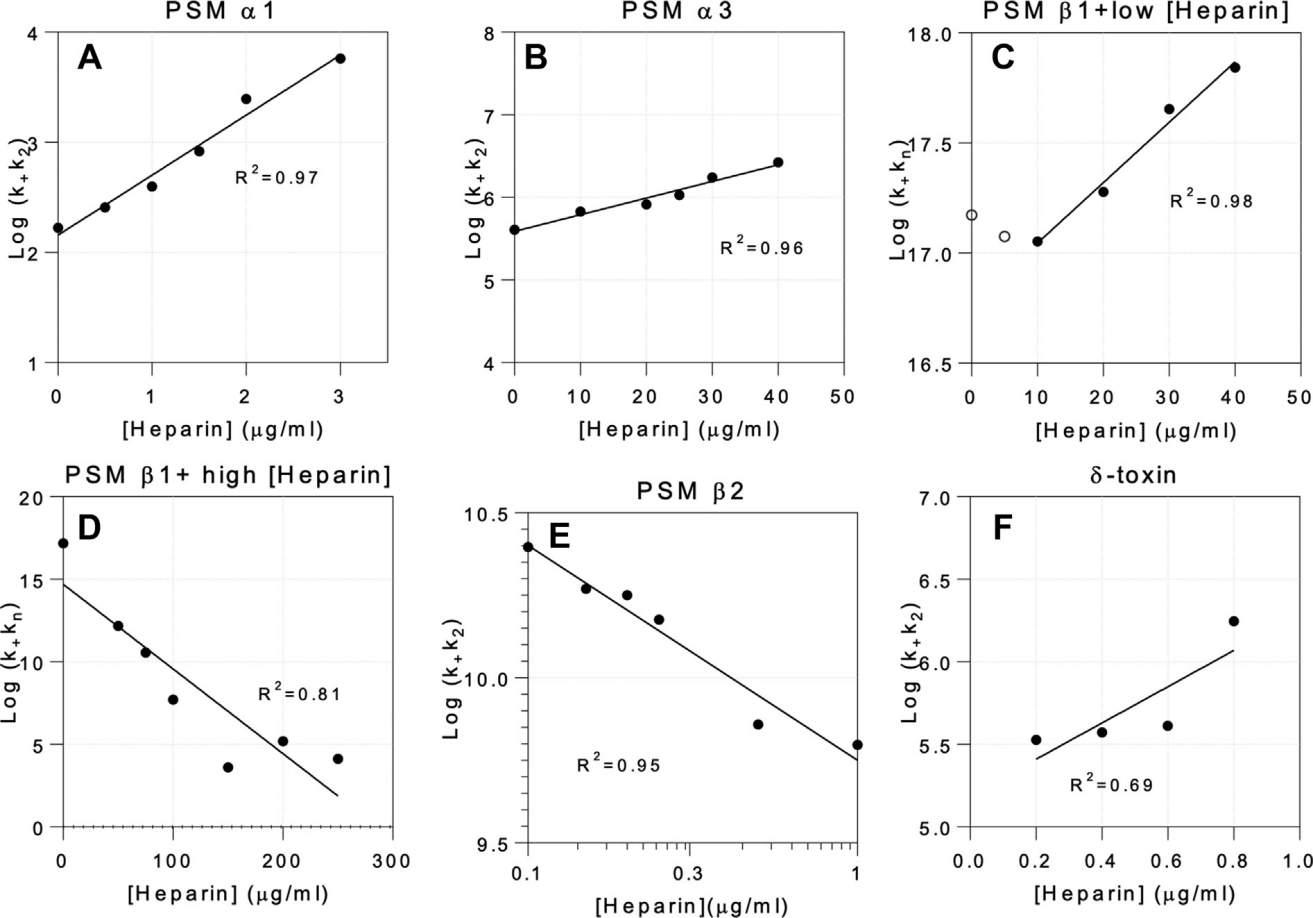
**Plots of different composite rate constants (obtained from fits to PSM aggregation data) *versus* heparin concentration.** (*A*) PSMα1, (*B*) PSMα3, (*C*) PSMβ1 at low heparin concentrations, (*D*) PSMβ1 at high heparin concentrations, (*E*) PSMβ2 (log–log plot), and (*F*) δ-toxin (showing poor linear correlation with heparin concentration). PSM, phenol-soluble modulin.

To establish how heparin affects the microscopic steps during the aggregation of PSMs, we turned to the program AmyloFit (28). Kinetic parameters from our previous analysis of PSMα1 aggregation in the absence of heparin (11) were used as fixed global parameters, whereas only one compound rate constant was individually fitted to each heparin concentration. This approach has previously been used for other amylogenic proteins to establish how, for example, inhibitors act on specific microscopic steps during aggregation (29, 30, 31). Fits to kinetic data are shown in Figure 2, and results from these fits are provided in Table 2 and Figure 3.

**Table 2:** Kinetic parameters of data fitting using the AmyloFit Web server in the presence of heparin using global constants for all parameters (*last row*) except the compound rate constants indiciated in columns 2, 4, 6, 8, and 10 which are fitted to individual heparin concentrations

[Heparin] (μg/ml)	PSMα1	[Heparin] (μg/ml)	PSMα3	[Heparin] (μg/ml)	PSMβ1	[Heparin] (μg/ml)	PSMβ1	Heparin (μg/ml)	PSMβ2
	*k*_+_*k*_2_		*k*_+_*k*_2_		*k*_+_*k*_*n*_		*k*_+_*k*_*n*_		*k*_+_*k*_2_
0	167	0	4.06 × 10^+5^	0	1.49 × 10^+17^	50	1.52 × 10^+12^	0.1	2.49 × 10^+10^
0.5	257	10	6.73 × 10^+5^	5	1.19 × 10^+17^	75	3.66 × 10^+10^	0.15	1.86 × 10^+10^
1	396	20	8.24 × 10^+5^	10	1.13 × 10^+17^	100	5.04 × 10^+7^	0.2	1.78 × 10^+10^
1.5	831	25	1.07 × 10^+6^	20	1.90 × 10^+17^	150	4.09 × 10^+3^	0.25	1.50 × 10^+10^
2	2.47 × 10^+3^	30	1.75 × 10^+6^	30	4.51 × 10^+17^	200	1.57 × 10^+5^	0.5	7.23 × 10^+9^
3	5.78 × 10^+3^	40	2.66 × 10^+6^	40	6.96 × 10^+17^	250	1.32 × 10^+4^	1	6.27 × 10^+9^
*m*_0_ = 110 μM *n*_c_ = 7.84 × 10^−6^ *k*_+_*k*_*n*_ = 6.98 × 10^−5^ (conc^−*n*2−1^ time^−2^) *n*_2_ = 0.00166 MRE: 0.00754		*m*_0_ = 96 μM *n*_c_ = 0.600 *k*_+_*k*_*n*_ = 257 (conc^−*n*2−1^ time^−2^) *n*_2_ = 0.123 MRE: 0.00570		*m*_0_ = 55 μM *n*_c_ = 3.92 *k*_+_*k*_2_ = 4.23 × 10^+3^ (conc^−*n*2−1^ time^−2^) *n*_2_ = 0.200 MRE: 0.00423		*m*_0_ = 55 μM *n*_c_ = 3.92 *n*_2_ = 0.2 *k*_+_*k*_2_ = 6.71 × 10^+5^ (conc^−*n*2−1^ time^−2^) MRE: 0.00905		*m*_0_ = 5.61 μM *n*_c_ = 0.572 *n*_2_ = 1.00 *k*_+_*k*_*n*_ = 0.0483 (conc^−*n*2−1^ time^−2^) MRE: 0.0117	

Abbreviation: MRE, mean squared residual error.

For PSMα1, we found the best fit when we allowed *k*_+_*k*_2_ to vary and restricted *k*_+_*k*_*n*_, *n*_c_, and *n*_2_ to the heparin-free values (Fig. 2*A*). Since *k*_+_*k*_*n*_ was kept constant during the data fitting, the kinetic parameter within the compound parameter *k*_+_*k*_2_ mostly affected by the presence of heparin is expected to be *k*_2_. Interestingly, log *k*_+_*k*_2_ values increase in a linear fashion when plotted against [heparin] (Fig. 3*A*). Similar linear relationships are seen in plots of the log of unfolding rate constants *versus* denaturant (urea and guanidinium chloride) (32) or surfactant (SDS) concentrations (33). While denaturants are present at molar concentrations (>100 mg/ml) and rely on weak interactions with the protein, SDS effects are seen at low millimolar (∼1 mg/ml) concentrations and are ascribed to high affinities (driven by electrostatics) and clustering on the protein (driven by hydrophobic effects). Given the low concentrations of heparin used (∼1 μg/ml), these interactions are clearly strong and may also be cooperative in nature.

Following this pattern, the best AmyloFit fits of PSMα3 aggregation in heparin were also obtained by varying *k*_+_*k*_2_, indicating that *k*_2_ is most affected as is the case for PSMα1 (Fig. 2*B* and Table 2). This leads to a similar semilog relationship (Fig. 3*B*), although the slope is reduced by a factor of ∼30 (slope of semilog plots: ∼0.54 for PSMα1 and 0.02 for PSMα3), indicating a somewhat weaker heparin–PSMα3 interaction.

PSMβ1–heparin interactions follow two different modes below and above 50 μg/ml heparin and are accordingly fitted as two separate sets of data in AmyloFit. Data with 0 to 40 μg/ml heparin are best fitted when *k*_+_*k*_*n*_ values are allowed to vary while keeping the other kinetic parameters *n*_c_, *n*_2_, and *k*_2_*k*_+_ constant (Fig. 2*C*). The latter indicates that *k*_+_ can be considered constant so that heparin mainly affects *k*_*n*,_ that is, primary nucleation. The value for *k*_*n*_*k*_+_ remains constant up to ca. 10 μg/ml heparin, after which it increases in a semilog linear manner (Fig. 3*C*). Data with 50 to 250 μg/ml heparin can be fitted by allowing *k*_*n*_*k*_+_ to vary with [heparin] while maintaining a single (global) fit value for *k*_+_*k*_2_ (Fig. 2*D*). The values of *k*_*n*_*k*_+_ decrease dramatically with [heparin], which we again attribute to a reduction in *k*_*n*_ since *k*_+_ is constant. This occurs in a linear manner when log (*k*_*n*_*k*_+_) is plotted *versus* [heparin] (Fig. 3*D*).

In the absence of heparin, PSMβ2 follows a primary nucleation- and elongation-dominated mechanism (11). However, in the presence of heparin, the best fit is obtained using a secondary nucleation–dominated aggregation model where *k*_+_*k*_*n*_ is kept a global constant and *k*_+_*k*_2_ is allowed to vary (Fig. 2*E*). As with PSMβ2, linearity is only seen when data are plotted in a log–log plot (Fig. 3*E*). Such log–log linearity is also seen, for example, ligand-binding or protonation/deprotonation systems, that is, strong and specific interactions. Heparin hence induces secondary nucleation in PSMβ2 while decreasing the aggregation kinetics by inhibiting the peptide's primary nucleation process (Fig. 2*E* and Table 2). This is also reflected in the value of *k*_*n*_*k*_+_, which is three orders of magnitude lower in the presence of heparin (0.0483 M^−nc^h^−2^) than its absence (48.8 M^−nc^h^−2^).

δ-toxin on its own did not show any ThT fluorescence increase, whereas heparin dramatically increased its ThT intensity. Since we did not have parameters for heparin-free aggregation, we fitted the data satisfactorily using a secondary nucleation–dominated aggregation mechanism with *k*_*n*_*k*_+_ as global fit and *k*_+_*k*_2_ as individual fits for each heparin concentration (Fig. 2*F*). *k*_+_*k*_2_ increases with increasing heparin but in a poorly linear manner (Fig. 3*F*).

It should be noted that fits with better mean squared residual error values can be obtained for the kinetic data for PSM peptides in the presence of heparin if the kinetic parameters are set to global fit instead of held as global constants (Fig. S2 and Table S1). This is to be expected since this allows more degrees of freedom during the data fitting. However, we find it most consistent to maintain parameters obtained from our previous experiments.

### Heparin has modest effects on the secondary structure and thermal stability of PSM fibrils

**Figure 4:**
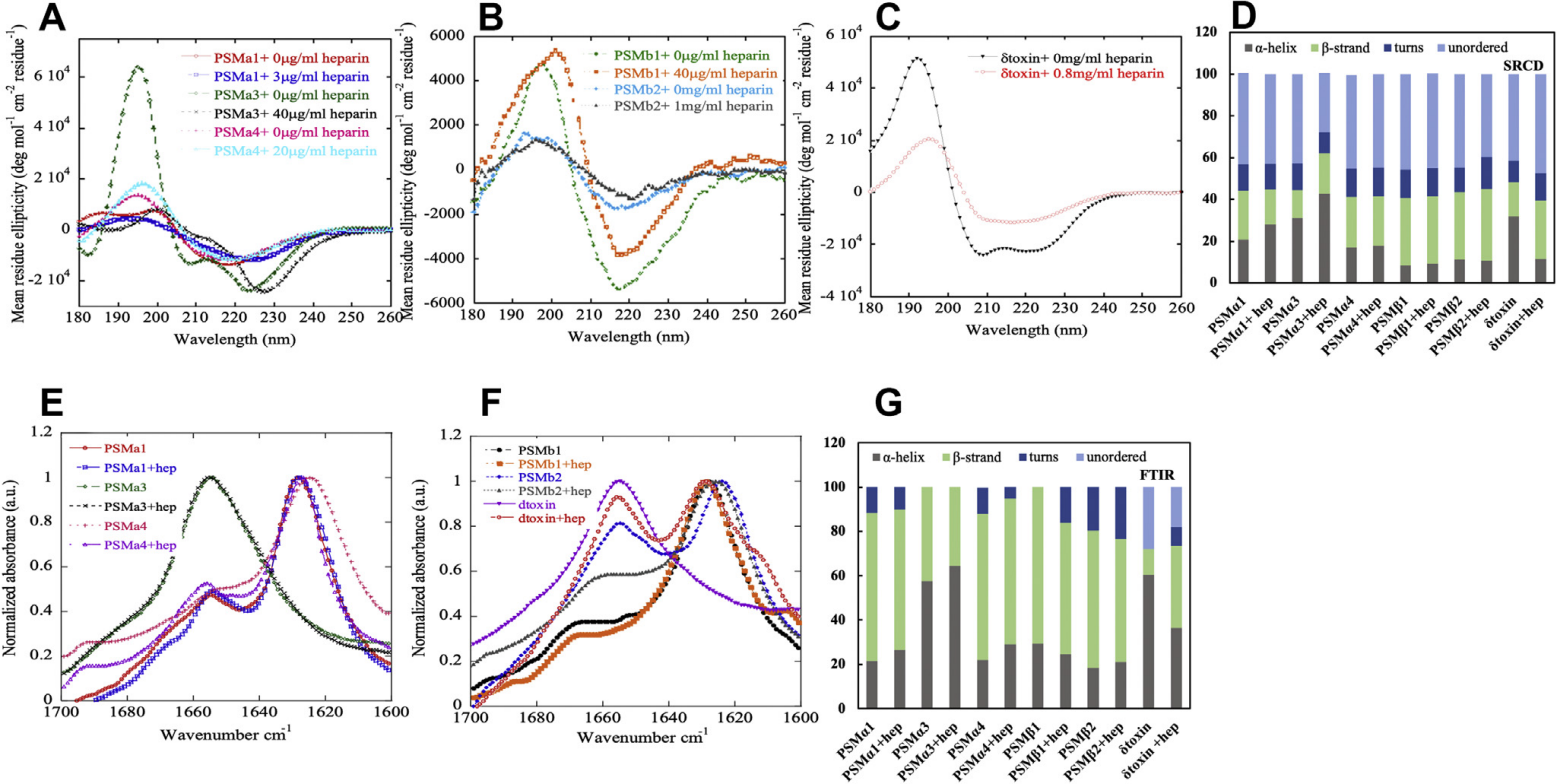
**Structural comparison of fibrils formed by different PSMs in the absence and presence of heparin.***A*–*C*, synchrotron radiation (SR) Far-UV CD spectra of all PSM fibrils incubated with or without heparin. Fibrillated samples were centrifuged (13,000 rpm for 30 min), supernatant discarded, and the pellet resuspended in the same volume of milliQ water. *D*, deconvolution of the SRCD spectra from panels *A*–*C*. *E* and *F*, FTIR spectroscopy of the amide I’ region (1600–1700 cm^−1^) of PSM fibrils formed in the absence and presence of heparin. PSMα1, PSMα4, PSMβ1, and PSMβ2 show a peak at 1625 cm^−1^ corresponding to rigid amyloid fibrils. In contrast, PSMα3 and δ-toxin show main peaks at 1655 cm^−1^, with the latter indicating more disordered fibrils. *G*, deconvolution of the FTIR spectra from panels *E* and *F*. PSM, phenol-soluble modulin; SRCD, synchrotron radiation CD.

Next, we addressed whether heparin affected the secondary structures of the fibrillar aggregates. For this, we turned to synchrotron radiation CD (SRCD) and attenuated total internal reflection (ATR)–FTIR spectroscopy. Each CD and FTIR spectrum was deconvoluted using the DichroWeb server (34, 35) and the OPUS 5.5 software (Bruker), respectively. Individual CD spectra are shown in Figure 4, *A*–*C* and with deconvolutions in Figure 4*D*, and FTIR spectra are presented in Figure 4, *E* and *F* with deconvolution results in Figure 4*G*.

In the absence of heparin, most fibrillar aggregates displayed a single minimum typical of β-sheets as seen for PSMα1 (218 nm), PSMα4 (218 nm), and PSMβ1 and PSMβ2 (220 nm). These peak positions are in good agreement with previous findings (36). The FTIR spectra of fibrils related to these four peptides were found to be very similar, with a well-defined intense peak at ∼1625 cm^−1^ indicative of amyloid β-sheet and a minor shoulder at ∼1655 cm^−1^ indicative of α-helical conformation (Fig. 4, *A*–*C*, *E*, and *F*).

PSMα3 aggregates in the presence and absence of heparin show cross-α-helical structure in both SRCD and FTIR spectra, in good agreement with previous reports (9, 37). The SRCD and FTIR spectra for PSMα4 in the absence and presence of heparin were internally consistent (Fig. 4, *A* and *E*). An FTIR peak around ∼1690 cm^−1^ suggests antiparallel β-sheets. SRCD spectra of PSMβ1 and PSMβ2 heparin-free aggregates are similar to those in the presence of heparin (Fig. 4, *B* and *F*). Furthermore, SRCD and FTIR spectra clearly indicate that heparin favors amyloid formation in δ-toxin, and significant secondary structural changes were observed in the presence of various concentrations of heparin (Fig. 4, *C* and *F*).

The thermal stability of the PSM aggregates in the absence and presence of heparin was evaluated by CD thermal scans (Fig. S3). Neither PSMα1 nor PSMα4 nor β-PSMs fibrils undergo any significant loss in signal up to 95 °C, indicating a thermally stable β-sheet structure. However, PSMα3 fibrils are thermally unstable under both conditions in the absence and presence of heparin, as a loss of structure is seen above 50 °C. Hence, heparin does not change the thermal stability of the fibrils.

### Heparin shows variable effects on the fibril morphology of PSMs

**Figure 5:**
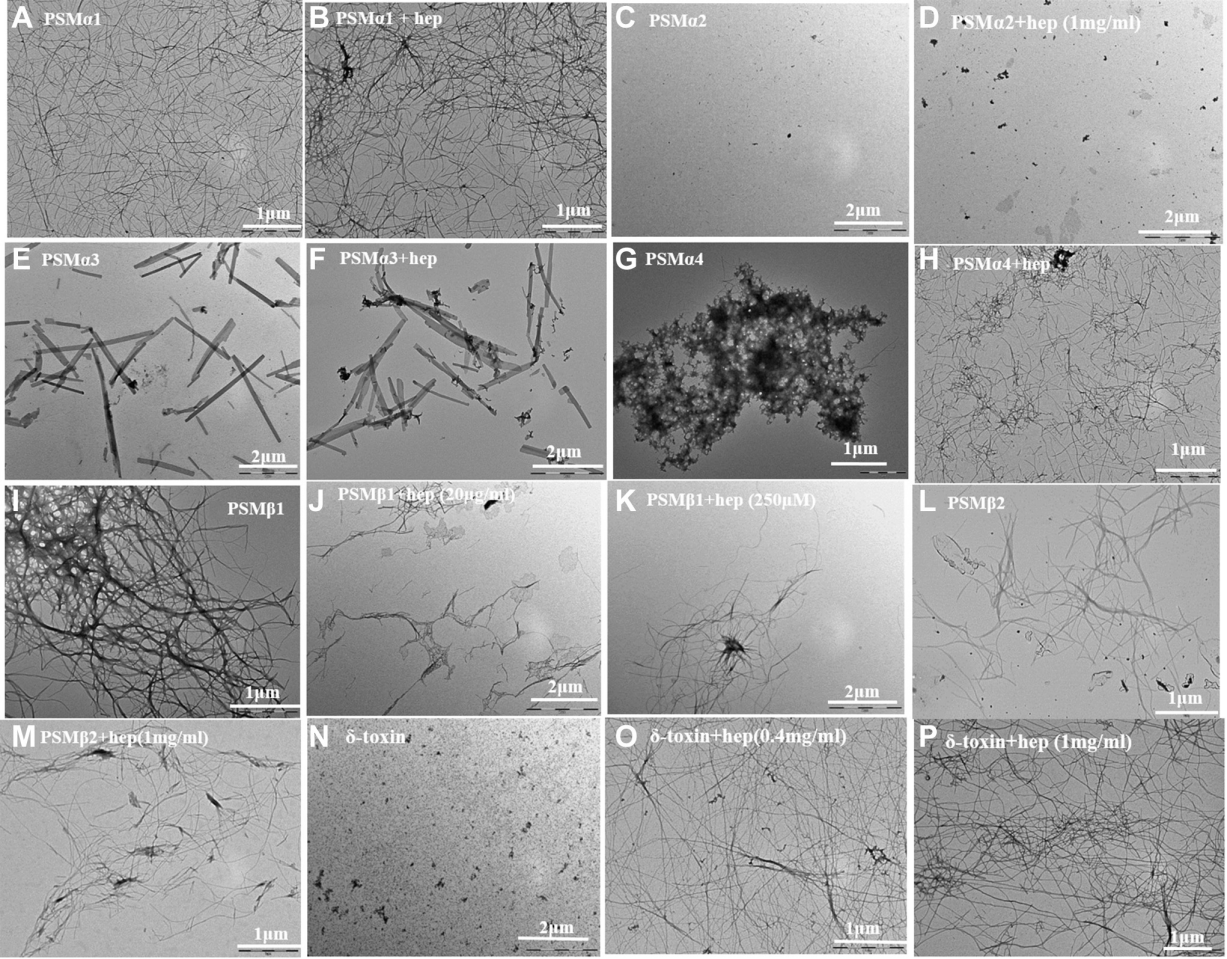
**Electron microscope images of fibrils formed from PSMs in the absence and presence of heparin.** TEM micrographs of (*A*) PSMα1 without heparin, (*B*) PSMα1 with 3 µg/ml heparin, (*C*) PSMα2 without heparin, (*D*) PSMα2 with 1 mg/ml heparin, (*E*) PSMα3 without heparin, (*F*) PSMα3 with 40 µg/ml heparin, (*G*) PSMα4 without heparin, (*H*) PSMα4 with 20 µg/ml heparin, (*I*) PSMβ1 without heparin, (*J*) PSMβ1 with 40 µg/ml heparin, (*K*) PSMβ1 with 250 µg/ml heparin (*L*) PSMβ2 without heparin, (*M*) PSMβ2 with 1 mg/ml heparin, (*N*) δ-toxin without heparin, (*O*) δ-toxin with 0.4 mg/ml heparin, and (*P*) δ-toxin with 1 mg/ml heparin. Note that scale bars vary between panels. PSM, phenol-soluble modulin; TEM, transmission electron microscopy.

The morphologies of the peptide fibrils in the absence and presence of heparin were analyzed by transmission electron microscopy (TEM). TEM analysis of PSMα1 incubated in the absence and presence of heparin showed amyloid-like fibrils in both cases (Fig. 5, *A* and *B*) though fibrils prepared with heparin were slightly thicker. No aggregated species were observed for PSMα2 (Fig. 5, *C* and *D*) in both conditions, consistent with the lack of increase in ThT fluorescence upon incubation with and without heparin. In the absence of heparin, PSMα3 formed long rod-like fibrils (Fig. 5*E*), which in the presence of heparin shows very similar fibrillar structure (Fig. 5*F*).

Heparin also encouraged formation of PSMα4 fibrils (Fig. 5, *G* and *H*). On their own, PSMα4 displays very thin fibrils visible at higher magnification with some distribution of spherical aggregates organized into small clusters on the grid, which upon incubation with heparin show nicely separated fibrillar structure (Fig. 5, *G* and *H*). PSMβ1 on its own formed highly ordered arrays of laterally associated fibers (Fig. 5*I*), which with heparin changed to disentangled thin aggregates (Fig. 5, *J* and *K*). We do not observe significant morphological difference between PSMβ2 fibrils obtained in the absence and presence of heparin (Fig. 5, *L* and *M*). Finally, TEM of δ-toxin confirmed the aggregation potential of heparin. While there were no visible fibrils in δ-toxin on its own, heparin led to large networks of thin fibers (Fig. 5, *N*–*P*).

### Interactions between heparin and PSM residues analyzed by peptide arrays

**Figure 6:**
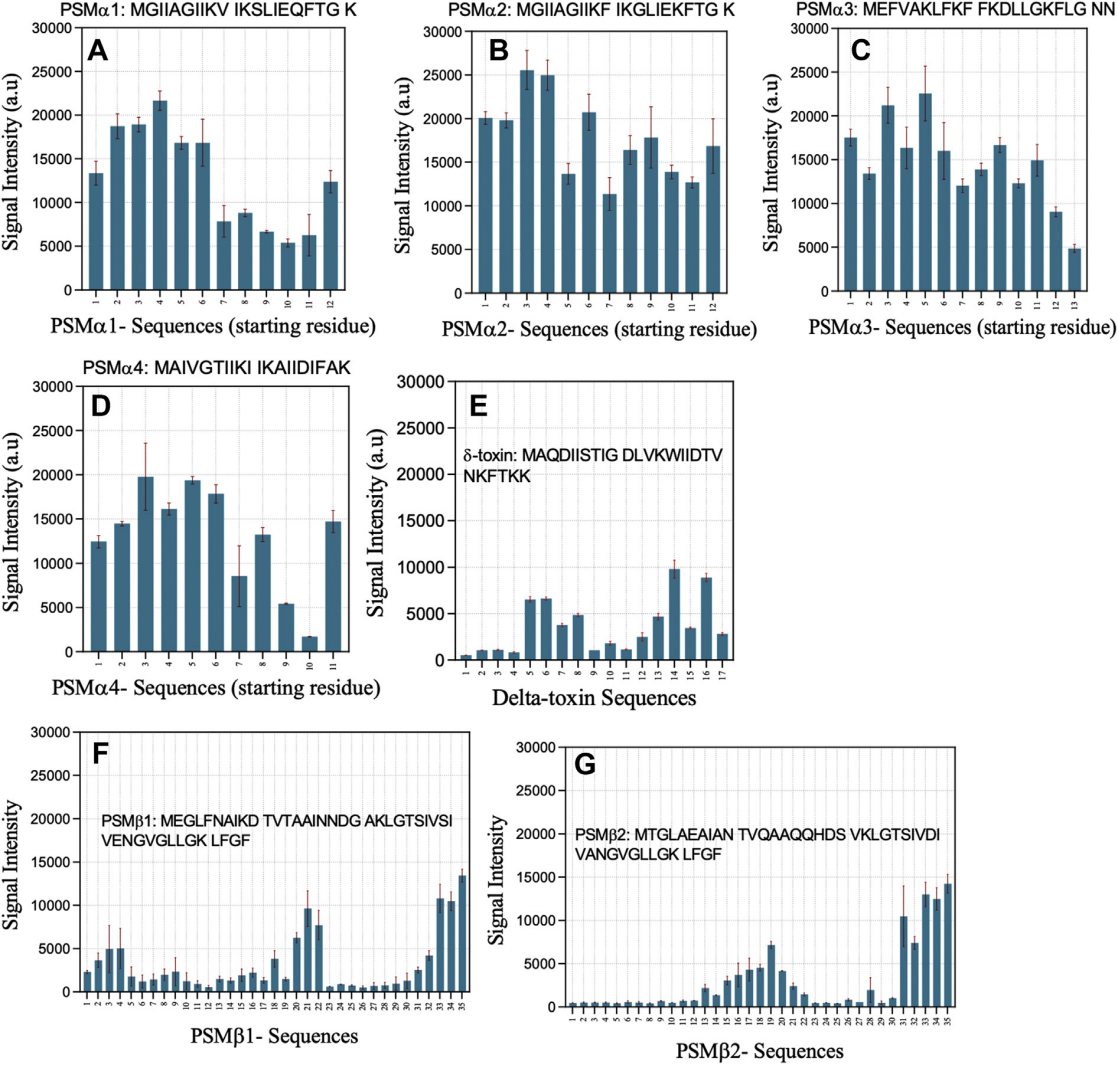
**Interaction of fluorescein-labeled heparin with different PSM peptides displayed on a peptide array.** Data provide signal intensity from different PSM sequences interacting with heparin. Full PSM sequences are provided in each panel. (*A*) PSMα1, (*B*) PSMα2, (*C*) PSMα3, (*D*) PSMα4, (*E*) δ-toxin, (*F*) PSMβ1, and (*G*) PSMβ2. For each spot, the number on the *x*-axis gives the residue position in the intact PSM sequence, corresponding to the starting residue in the spot's 10-mer peptide. PSM, phenol-soluble modulin.

We explored the interaction between fluorescein-labeled heparin and PSM sequences using a peptide array chip displaying 10-residue immobilized peptides in staggered arrangements. Thanks to the fluorescein label, it was possible to quantitate the amount of heparin bound to each peptide fragment (Fig. 6). All α-type PSMs bind more heparin than β-PSM, but the intensity is not equally distributed along the length of each PSM. High heparin affinity is shown by peptides corresponding to the N-terminal half of α-PSMs and the middle and C-terminal part of β-PSMs. We attempted to probe possible correlations between signal intensity and the peptides' physical–chemical characteristics such as charge and hydrophobicity (38). As shown in Table 3, the sequence charge is the most significant contributor, especially for β-PSMs, with higher positive charge leading to higher signal intensities. This is to be expected in view of heparin's highly anionic nature.

**Table 3:** *p* Values from multiple regression analysis of the correlation between signal intensity and the two parameters charge and hydrophobicity

Peptide	Charge	Hydrophobicity
PSMα1	++^a^	+^b^
PSMα2	+^b^	+^b^
PSMα3	++^a^	–c
PSMα4	+^b^	–c
PSMβ1	+++^d^	+^b^
PSMβ2	+++^d^	+^b^

Data are based on peptide arrays of wildtype PSM sequences.

a 0.0001 > *p* value > 0.001.

b 0.001 > *p* value > 0.05.

c *p* value > 0.05.

d 0.0001 > *p* value.

Note however that *a priori* we cannot predict whether high binding by heparin would promote aggregation (*e.g.*, by forming a template for the extended state, leading to amyloid) or inhibit it (by sequestering monomers from interacting with other monomers). According to our ThT assays (Fig. 1), heparin accelerates the fibrillation of PSMα1, PSMα3, PSMα4, δ-toxin, and PSMβ1 at low heparin concentrations, whereas it inhibited PSMβ1 at high heparin concentrations as well as PSMβ2. We conclude from this that the high affinity of heparin to N termini of α-PSM promotes fibrillation, whereas binding to the middle and C-terminal regions of β-PSMs inhibits fibrillation.

**Figure 7:**
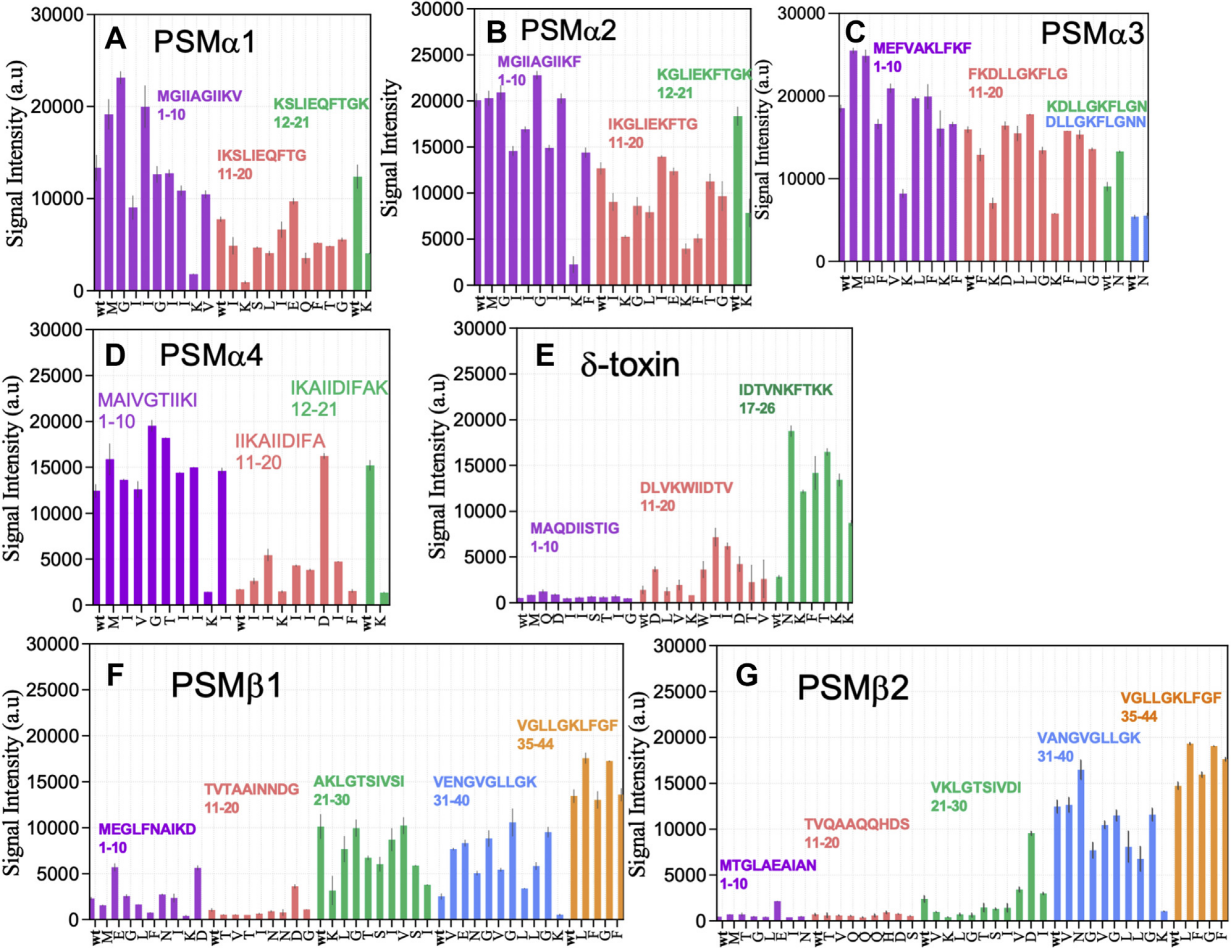
**Interaction of fluorescein-labeled heparin with peptide-array sequences designed for Ala scans of different PSMs.** Data provide signal intensity from different Ala-scanned PSM sequences interacting with heparin. Each PSM peptide was divided into 10-residue sequences, each with their own color; in each peptide, positions were individually replaced by Ala from left to right. “wt” is the initial peptide before starting Ala scan. Each letter on the *x*-axis shows the residue that is replaced by Ala, (*A*) PSMα1, (*B*) PSMα2, (*C*) PSMα3, (*D*) PSMα4, (*E*) δ-toxin, (*F*) PSMβ1, and (*G*) PSMβ2. PSM, phenol-soluble modulin.

To elucidate the role of individual residues in the heparin interaction, we carried out an Ala scan of all PSMs (data for all these scans are provided in Fig. 7).

In PSMα1, an *increase* in signal intensity compared with wildtype was caused by mutations M1A, G2A, and I4A (which maintain the same charge but lead to either a fall or a rise in hydrophobicity). A *decrease* in signal was caused by removal of positive charge (K9A, K12A, and K21A). This illustrates clearly how electrostatic interactions are the main (but not the only) drivers of heparin–peptide interactions.

Similarly, K-to-A mutations in PSMα2 and also in the rest of PSM peptides (including PSMα3, PSMα4, δ-toxin, PSMβ1, and PSMβ2) decreased heparin binding but so did loss of hydrophobicity: mutations I3A, I6A, and F18A for PSMα2 and I3A, I11A, and L14A for PSMα1 led to a 25 to 50% drop in intensity. We were unable to obtain ThT fibrillation curves for PSMα2; thus, we cannot conclude what effect binding would have on its fibrillation kinetics.

Moreover, removal of negative charge (*e.g.*, for E and D to A residues) leads to an increase in signal intensity, but mutations altering hydrophobicity had an even more marked increase in binding for peptides with the same charges (Table S2). Mutation M1A also led to increase in signal for PSMα3 and PSMα4 same as PSMα1. For PSMα3, K-to-A mutations led to the highest decrease in signal intensity, again emphasizing the role of charge. For PSMα4, except the mutations affect the charge, the truncation mutations T6A, I12A, I14A, I15A, and I17A increased binding as did the insertion mutation G5A. G-to-A/T mutations increase hydrophobicity, whereas I-to-A mutations decrease it; neither affect charge. Interestingly, the I-to-A mutation led to a drop in signal for PSMα1 (I4A and I11A) and PSMα2 (I3A, I4A, I6A, and I11A) but increased it for PSMα4 and δ-toxin (I16A and I17A), indicating different roles for Ile.

For δ-toxin, the Ala scan of first ten residues did not show major changes. For the second ten residues, almost all mutations led to higher signal intensity, and the most important mutations are D11A, W15A, I16A, I17A, and D18A. Ala scans of the last six residues increased the signal intensities to the highest level. Remarkably, this was also the case for three K-to-A mutations. Note that these six spots are the only positively charged spots in Ala scan of δ-toxin (which does not fibrillate in the absence of heparin), and they could be binding partners for heparin that promote fibril formation. Also in PSMβ1 and PSMβ2, removal of anionic Glu–Asp led to increased binding, whereas removal of Lys decreased signal intensity.

Table 4 shows that for PSMβ1 and PSMβ2, charge is most strongly correlated with binding (*p* = 6.12 × 10^−6^ and 3.01 × 10^−9^, respectively), whereas hydrophobicity has a much weaker effect on PSMβ1 (*p* = 0.025) and PSMβ2 (*p* = 0.313). Based on multiple regression analysis with two variants, charge and hydrophobicity, the predicted signal intensities nicely fit the measured signals, indicating the central importance of these two parameters in the interaction between PSMs and heparin (Fig. S4).

**Table 4:** *p* Values from multiple regression analysis of the correlation between signal intensity and the parameters charge and hydrophobicity

Peptide	Charge	Hydrophobicity
PSMα1	++^a^	
PSMα2	++^a^	++^a^
PSMα3	++^a^	–b
PSMα4	+++^c^	–b
PSMβ1	+++^c^	+^d^
PSMβ2	+++^c^	–b

Data are based on Ala scans of PSM peptides by peptide array.

a 0.0001 > *p* value > 0.001.

b *p* value > 0.05.

c 0.0001 > *p* value.

d 0.001 > *p* value > 0.05.

### Heparin promotes biofilm formation both in the presence and absence of PSMα/β

**Figure 8:**
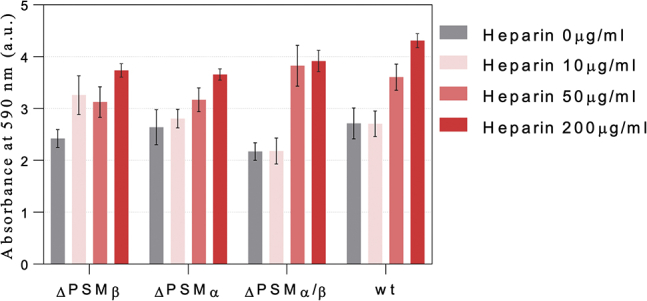
**Biofilm formation in the presence and absence of heparin based on crystal violet staining of biofilm**.

To put our observations in a biological context, we investigated how heparin affects biofilm formation. Accordingly, we incubated *S. aureus* Newman strain as a model of *S. aureus* human infections having a robust virulence phenotype and ability to form biofilm (39, 40) and three different PSM mutants of this strain with 10 to 200 μg/ml of heparin. Incubation of wildtype *S. aureus* with heparin increased the amount of biofilm significantly at >10 μg/ml heparin (Fig. 8). A strain that only produces PSMα (ΔPSMβ) shows a higher biofilm formation even at low [heparin] compared with ΔPSMα; this could be caused by the ability of heparin to induce PSMα fibrillation. However, an increase in the biofilm formation of ΔPSMα/β in the presence of heparin indicates that other mechanisms are also involved in biofilm formation.

## Discussion

*S. aureus* is an important human pathogen causing many different hospital- and community-associated infections. This is promoted by its ability to form biofilm, aided by amyloid-forming PSMs (41), and thus increase resistance to antibiotics (42). *S. aureus* is particularly prone to form biofilm on catheters; furthermore, heparin is commonly used as an anticoagulant in these catheters (17). This inspired us to investigate the effect of heparin on PSM fibrillation.

### Heparin shows a range of effects on aggregation, which are very sensitive to peptide sequence

Our study highlighted that heparin accelerates fibril formation for α-PSMs (PSMα1, PSMα3, PSMα4, and δ-toxin) at concentrations as low as 1 μg/ml (PSMα1), though the effect was generally seen in the range of 0.02 to 1 mg/ml. In contrast, heparin inhibits the fibrillation of PSMβ1 by decreasing the end-level ThT fluorescence intensity at low heparin concentrations (<50 μg/ml) and increasing the lag phase of PSMβ2s by >50 μg/ml heparin (Fig. 1). The interaction is mainly driven by electrostatic interactions between PSMs and heparin as seen for many other protein–heparin interactions (25, 26, 43).

In the PSMα family, heparin induces fibrillation by reducing nucleation time and enhancing the end-level ThT fluorescence intensity (Fig. 1, *A*–*D*). It also induces aggregation of the otherwise nonaggregating δ-toxin (Fig. 1*G*). PSMβ1 is inhibited in two different modes: below 50 μg/ml heparin, heparin inhibits the nucleation step but does not affect growth rates, but above 50 μg/ml heparin, increased growth rate and end-level ThT fluorescence intensity while increasing the lag phase (Fig. 1*E*). Heparin had mixed effects on PSMβ2, increasing the lag time as well as end-level ThT fluorescence intensity (Fig. 1*F*). It is remarkable that PSMβ1 and PSMβ2 show very distinct aggregation behavior despite their high similarity. PSMβ1 aggregates efficiently at very low concentrations and responds in a bimodal manner to heparin, with different behavior at low *versus* high heparin concentrations. AmyloFit analysis reveals that the *k*_+_*k*_2_ is mostly affected in the α-PSM peptide, whereas incubation with heparin leads to variations in *k*_+_*k*_*n*_ for β-PSM peptides, thus highlighting the effect of heparin on the nucleation phase of aggregation for the β-PSM peptides in particular (Fig. 2 and Fig. S2).

The marked increase in the final ThT level is also seen as a higher level of fibril formation. Similar effects have been reported for amyloidogenic proteins like α-synuclein and transthyretin (21, 23). Heparin copellets with the aggregates, showing strong binding and a possible templating role in fibrillation (21, 23, 26, 44). The shortening of the lag phase seen for PSMα1 and PSMα3 might be caused by heparin's induction of conformational changes favoring fibrillation process or by stabilizing early stage aggregates (14, 23, 45).

### Cationic heparin-binding motifs in the α-PSM family may drive fibrillation

Heparin-binding domains often contain a high proportion of positively charged Lys and Arg, which can interact with anionic glycosaminoglycans (14). These residues often occur as motifs with basic amino acids in close proximity like XBBBXXBX and XBBXBX sequences where B and X are basic and nonbasic residues, respectively (46, 47). The proximity of B residues likely leads to cooperative binding effects. Such motifs are found in the α-PSM family, for example, “KVIK” in PSMα1, “KLFK” in PSMα3, “KIIK” in PSMα4, and “KFTKK” in δ-toxin. For β-PSMs, the Lys residues are further from each other, and their net charge at pH 7 is overall negative. This might explain the difference in behavior. Our peptide array data also confirm the importance of positively charged residues (mainly Lys) whose replacement by Ala abolished heparin binding (Fig. 7). This is consistent with other studies. Removing positively charged residues increased the *t*_1/2_ of aggregation of human muscle acylphosphatase in the presence of heparan sulfate, highlighting the importance of Lys and Arg as binding sites for heparan sulfate (48). Furthermore, removal of negatively charged residues (D and E residues) increased the binding of heparin. Both confirm the importance of electrostatic interaction between PSMs and heparin. The role of complementary electrostatics is also confirmed by the observation that positively charged polysaccharides like chitosan do not increase biofilm formation (17, 49, 50). Alanine scan of peptides on peptide array indicated that charge is more important than hydrophobicity as K-to-A mutations increased the hydrophobicity but did not increase the binding of heparin. The structure of PSMα3 fibrils reveals that some Lys residues are not involved in intermolecular fibrillar contacts but are speculated to be related to the cytotoxicity of the peptide toward human cells (*e.g.*, through interactions with the membrane) as mutations of these residues to Ala result in reduced toxicity (37). This implies that these residues can take part in electrostatic interactions with heparin without interfering with amyloid formation.

Structural analysis of fibrils demonstrate typical β-sheet fibrils for PSMα1 (218 nm), PSMα4 (218 nm) and PSMβ1 and PSMβ2 (220 nm) as reported (11, 36, 51), and this is largely unaffected by heparin. Similarly, the characteristic but unusual cross-α fibrils of PSMα3 are maintained in the presence of heparin (9, 37). Heparin led the nonaggregating δ-toxin peptide (which forms α-helices in solution) to form β-sheet amyloid fibrils (Fig. 1*G*). All fibrils show high thermal stability except the cross-α PSMα3 fibrils, which are unstable above 50 °C (Fig. S3). Similar modest thermal stability has been reported for fibrils of PSMα3-LFKFFK segment (51). Thus, the cross-α structure may be inherently less stable than cross-β fibrils because of the difference in the type of intermolecular contacts.

Incubation of *S. aureus* Newman strain and its *psm* mutants in the presence of heparin show that biofilm formation was significantly promoted in all strains by heparin. There were differences in the strains' response at different heparin concentrations, but no clear picture emerges at this stage. We suspect that this may in part reflect that we measure the end point of fibrillation rather than monitoring biofilm formation in real time, which is currently nontrivial because of the scarcity of specific probes targeting functional amyloid.

In summary, our study uncovered a diversity of mechanistic effects of heparin on the fibrillation of PSMs. There were modest differences in the kinetics of the heparin-stimulated fibrillation reaction of PSMs, with the kinetics being fastest with PSMα3 and slowest with δ-toxin, and there are significant differences in the seven PSM peptides' affinities for heparin. While heparin promotes fibrillation of α-PSMs and δ-toxin, it inhibited but did not abolish β-PSM fibrillation (Fig. 1), consistent with its ability to promote *S. aureus* biofilm formation. Heparin mostly targets the nucleation step and thus the lag phase, whereas increasing end-level ThT fluorescence intensity suggest higher levels of fibrillation (Fig. 3 and Fig. S2). Furthermore, our data demonstrate that positively charged residues close to each other in α-PSMs and δ-toxin provide suitable region to stabilize binding of the highly negatively charged heparin. In addition, in contrast to most previous studies showing only the effects of heparin as a promoter of fibrillation (21, 52), our results demonstrated that heparin has a dual effect, and it acts as an inducer or an inhibitor in the fibrillation of PSMs, which may contribute both to the integrity and dynamics of formation of biofilms.

## Experimental procedures

### Peptides, reagents, and solutions

The peptides PSMα1 (MGIIAGIIKVIKSLIEQFTGK), PSMα2 (MGIIAGIIKFIKGLIEKFTGK), PSMα3 (MEFVAKLFKFFKDLLGKFLGNN), PSMα4 (MAIVGTIIKIIKAIIDIFAK), PSMβ1 (MEGLFNAIKD TVTAAINNDG AKLGTSIVSI VENGVGLLGK LFGF), PSMβ2 (MTGLAEAIAN TVQAAQQHDS VKLGTSIVDI VANGVGLLGK LFGF), and δ-toxin (MAQDIISTIG DLVKWIIDTV NKFTKK) were purchased from GenScript Biotech. All peptides were N-terminally formylated with a purity of >95%. All reagents and chemicals were of analytical grade. Heparin–fluorescein conjugate and chemicals including hexafluoroisopropanol, ThT, TFA, and crystal violet solution (2.3%) were from Sigma–Aldrich, Ltd. Heparin (catalog no. Y0001282) was from European Pharmacopoeia. Dimethyl sulfoxide was from Merck. Peptide stock solutions were filtered using polyvinylidene fluoride 0.22 μm syringe filters (Millex-HV; Millipore) before use.

### Peptide pretreatment

For aggregation kinetics and secondary structure analysis (CD and FTIR), each PSM peptide stock was pretreated to disassemble any preformed aggregates. All seven dry lyophilized peptides (PSMα1–4, PSMβ1 and PSMβ12, and δ-toxin) were freshly dissolved to a final concentration of 0.5 mg/ml in hexafluoroisopropanol:TFA (1:1 v/v) and sonicated for 5 × 20 s with 30 s intervals using a probe sonicator, followed by incubation at RT for 1 h. Solutions were then aliquoted out, and organic solvent was evaporated using a speedvac (1000 rpm for 3–4 h) at RT. Dried peptide stocks were stored at −80 °C prior to use.

### ThT fibrillation assay

About 10 mg of heparin was dissolved in 1 ml milliQ water and passed through a polyvinylidene fluoride 0.45 μm syringe filter. PSMs were thawed and dissolved in dimethyl sulfoxide to 10 mg/ml prior to use. All seven freshly prepared peptides were diluted (typically to 0.25–1 mg/ml) into sterile milliQ water containing 40 μM ThT with 0 to 1 mg/ml heparin in a final volume of 100 μl in a 96-well black polystyrene microtiter plates. Different PSMs with a fixed monomeric peptide concentration (0.25–1.0 mg/ml) were supplemented with appropriate amounts of heparin (0–1 mg/ml) for the entire study. Heparin itself did not give rise to any fibrillation in the presence of ThT (data not shown). Because of the very low background fluorescence of ThT in buffer, this signal was not subtracted from sample data. ThT fluorescence was monitored on a Fluostar Omega (BMG Labtech) plate reader in bottom reading mode at 37 °C under quiescent conditions. The plate was sealed with metal sealing tape to prevent evaporation. ThT fluorescence of all PSMs except PSMα3 was measured every 10 min with an excitation filter of 450 nm and an emission filter of 482 nm under quiescent conditions. For PSMα3, ThT fluorescence was measured every 20 s with an excitation filter of 450 nm and an emission filter of 482 nm. About 0 to 1 mg/ml heparin alone was tested in separate experiments. All measurements were in triplicate.

### SRCD spectroscopy

SRCD spectra of PSM fibrils were collected at the AU-CD beam line of the ASTRID2 synchrotron, Aarhus University. PSM samples aggregated in the absence and presence of heparin were collected directly from the 96-well plates and pelleted at 13 krpm for 30 min. The supernatant was gently removed from each sample, and the pellet fraction was resuspended in milliQ water. Three to five successive spectra of fibrillated PSMs (in the absence and presence of heparin) were recorded from 280 to 170 nm in a 0.2-mm path length cuvette with a dwell time of 2 s at 1-nm intervals at 25 °C. All SRCD spectra were processed, and their respective averaged baseline (a solution containing all components of the sample, except the protein) subtracted, smoothing with a 7-point Savitzky–Golay filter. The secondary structural content of individual SRCD spectra of PSM fibril samples (in the absence and presence of heparin) was determined using DichroWeb server (34, 35). Each spectrum was fitted using three different analysis programs (Selecon3, Contin, and CDSSTR) with the SP175 reference dataset (53). An average of the structural component contributions from the three analysis programs was used.

### Thermal fibril stability by CD analysis

Thermal CD spectra were recorded on a JASCO-810 (Jasco Spectroscopic, Co, Ltd) spectrophotometer equipped with a Peltier thermally controlled cuvette holder. At the end of ThT kinetics experiments, individual triplicate samples fibrillated in the absence and presence of the maximum concentrations of heparin, that is, 3 μg/ml for PSMα1, 40 μg/ml for PSMα3, 50 μg/ml for PSMα4, 250 μg/ml for PSMβ1, 1 mg/ml for PSMβ2, and 1 mg/ml for δ-toxin were pelleted at 13 kpm for 30 min and the supernatant was removed. The remaining pellets were resuspended in the same volume of milliQ water, and thermal scans were recorded at 220 nm from 25 to 95 °C with a step size of 0.1 °C.

### ATR–FTIR spectroscopy

PSM fibrils were prepared as aforementioned. About 5 μl of the sample was applied on the surface of the ATR module and dried under a steam of nitrogen gas. FTIR spectra were recorded on a Tensor 27 FTIR instrument (Bruker Optics, Inc) equipped with an ATR accessory with a continuous flow of nitrogen gas. Measurements were performed as an accumulation of 64 scans with a spectral resolution of 2 cm^−1^ over a range of 1000 to 3998 cm^−1^. Atmospheric compensation and baseline correlation was executed. The individual components of the spectrum were determined through second derivative analysis of curves by employing OPUS 5.5 software. For comparative studies, all absorbance spectra were normalized.

### TEM

The morphology of the PSM species in the presence and absence of heparin was analyzed with TEM. About 5 μl of end-point samples from the nonshaking ThT assay of each PSM was transferred to carbon-coated formvar electron microscopy grids followed by 2 min of incubation at RT. Buffer was then removed by blotting the grid with Whatman filter paper, washed with 5 μl milliQ water, and stained with 1% uranyl acetate for 2 min, after which excess staining solution was removed with filter paper. Finally, the grids were washed twice with 5 μl of milliQ and dried before analysis. Samples were viewed in a Morgagni 268 FEI Phillips Electron microscope equipped with a charge-coupled device digital camera, operated at 80 kV.

### Heparin–PSM interactions measured using a peptide array

To probe interactions between heparin and PSMs, 351 different 10-residue peptides corresponding to different parts of the PSM sequences were immobilized on a microarray chip (full list provided in Tables S3 and S4) and incubated with fluorescein-labeled heparin (54). In this array, each new peptide constituted a 10-residue window of a given PSM sequence shifted forward by one residue compared with the preceding peptide, giving a nine-residue overlap. For Ala scanning, each residue in a given 10-residue sequence was consecutively replaced by Ala before moving on to the next 10-residue peptide. As part of this procedure, the microarray was first blocked in a solution containing 3% (w/v) whey protein in Tris saline buffer with 0.1% Tween-20 (TSB-T) incubated overnight at 4 °C and washed three times with TSB-T. Subsequently, it was incubated with fluorescein-labeled heparin (diluted to 0.05 mg/ml in PBS) for 4 h at RT. The microarray was washed three times with TSB-T, air dried in the dark, and scanned using a Typhoon Trio scanner (GE Life Sciences). Dot intensities in the scanned image were quantified using ImageJ (https://imagej.nih.gov/ij/).

### Biofilm measurements with crystal violet

*S. aureus* (strain Newman) and three deletion mutants, ΔPSMα, ΔPSMβ, and ΔPSMα/β, were grown on an LB plate overnight. A single colony was transferred to TSB medium, grown up overnight at 37 °C and then adjusted to an absorbance of ∼0.5 and further diluted 1:100 in fresh 3.3 g/l peptone, 2.6 g/l NaCl, and 3.3 g/l glucose media. Heparin was added (from a 5 mg/ml stock in milliQ water) to the desired concentration, and 100 μl was then incubated in a 96-well plate for 24 h at 37 °C with mild shaking (50 rpm). The solutions were gently removed from all wells, after which the biofilm was washed once with PBS and air dried for 30 min at RT. About 100 μl of crystal violet solution of 2.3% (Sigma) was added to all wells, incubated at RT for 10 min, and removed. All wells were washed twice with PBS. The plate was air dried for 30 min, after which 150 μl of 33% (v/v) acetic acid was added to each well to release the dye attached to the biofilm. Finally, the absorbance of the released crystal violet was measured at 590 nm.

## Data availability

The authors will make all data underlying the findings described in the article fully available upon request by the American Society for Biochemistry and Molecular Biology. In principle, all data can be read from the figures but will in some cases be beyond current graphical resolution; raw data can be provided.

## Supporting information

This article contains supporting information.

## Conflict of interest

The authors declare that they have no conflicts of interest with the contents of this article.
