# Supplementary material for: Heparin promotes fibrillation of most phenol-soluble modulin virulence peptides from Staphylococcus aureus
Source: J Biol Chem. 2021 Jul 14;297(2):100953. doi: 10.1016/j.jbc.2021.100953 (PMC8363829; doi:10.1016/j.jbc.2021.100953)
Supplement: Supplemental Figures S1–S4 and Tables S–S4 [file mmc1.pdf]

Supplementary Information

Heparin promotes fibrillation of most phenol soluble modulin peptides from *S. aureus*: a possible strengthening of the bacterial biofilm

Zahra Najarzadeh, Masihuz Zaman, Vita Sereikaite, Kristian Strømgaard, Maria Andreasen and Daniel E. Otzen

Figure S1

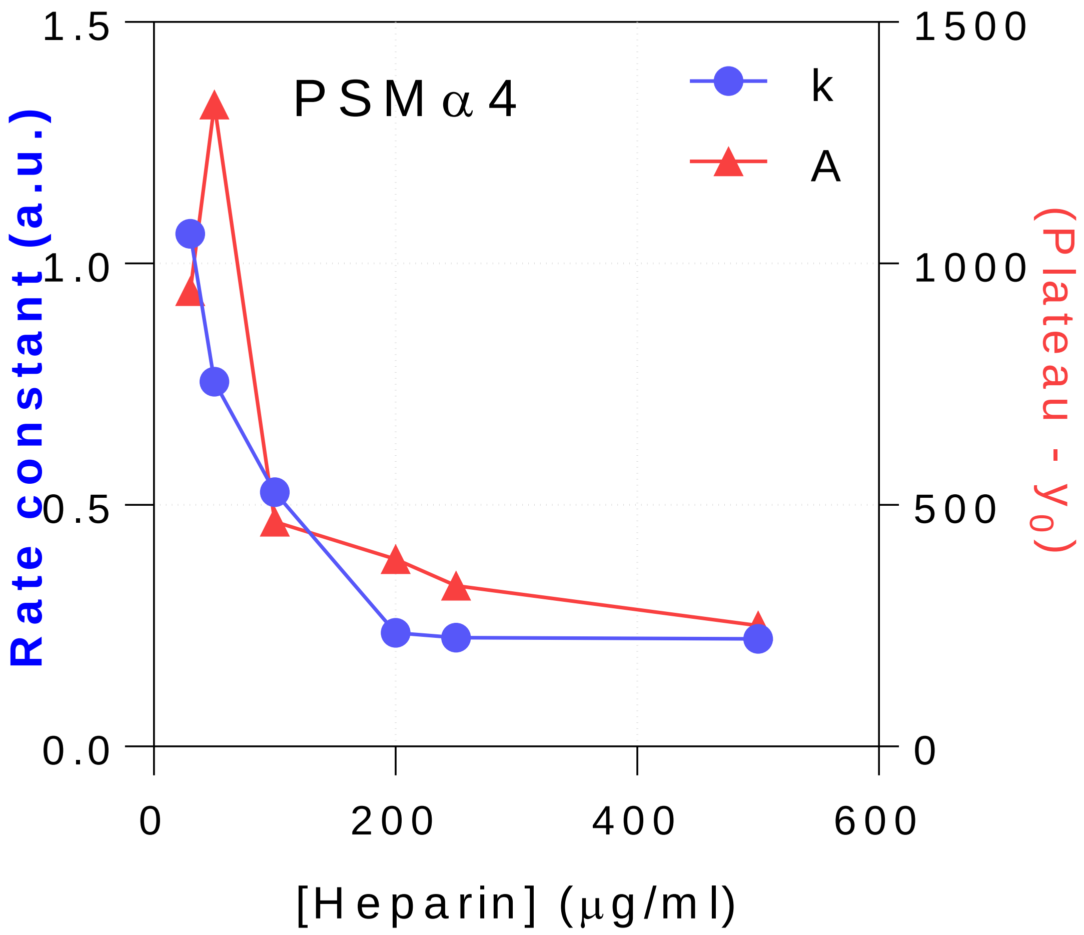

Figure S1: Kinetic parameters for PSMα4 versus heparin concentration. Data for 30-500 µg/mL heparin in panel D were fitted with an exponential decay to yield rate constant *k* and amplitude *A*.

Figure S2

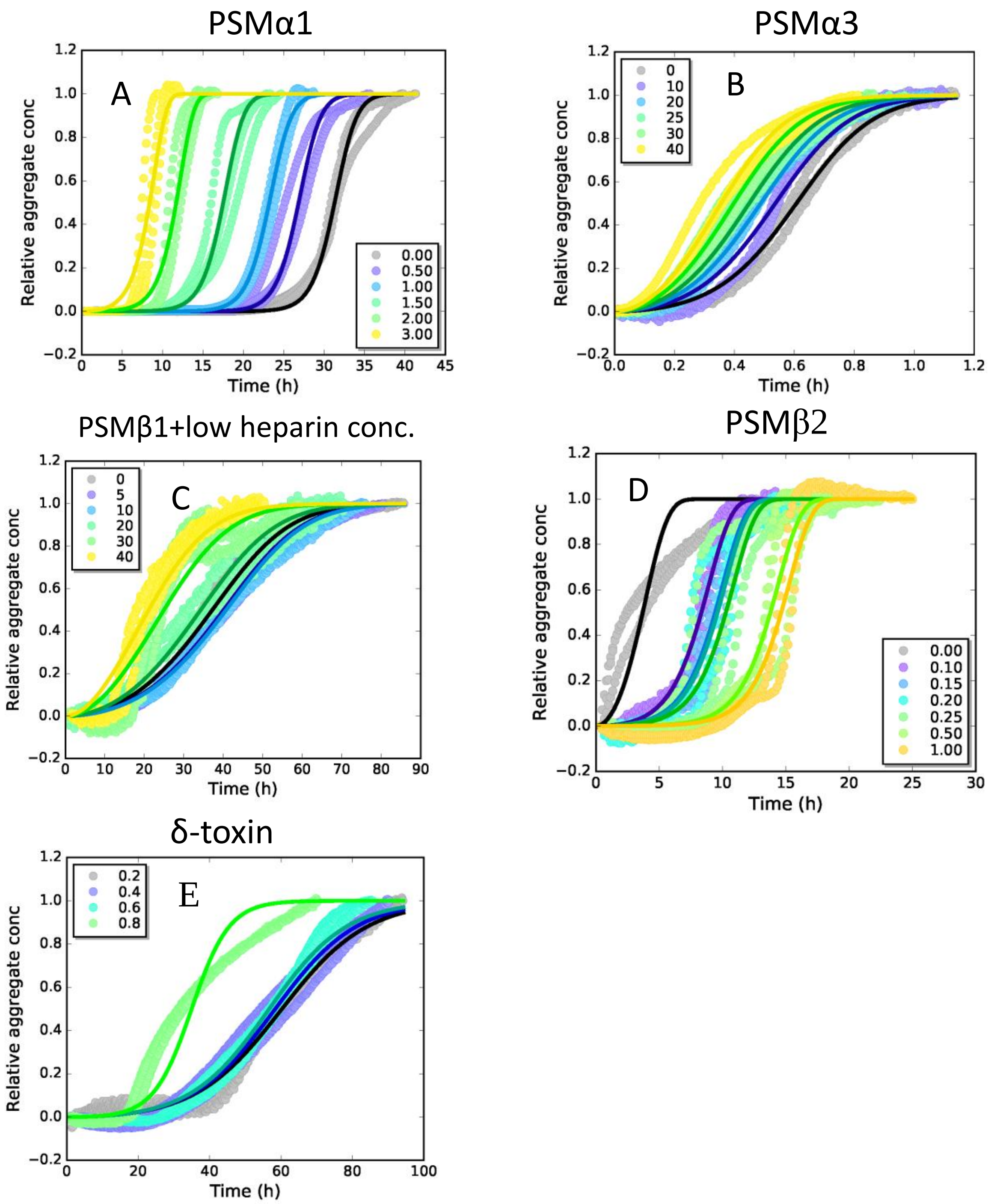

Figure S2: Fitting of aggregation kinetic data for PSM peptides in the presence of heparin. The heparin concentration in µg/mL is indicated for each curve, all data fitted to a secondary nucleation dominated model using global fitting of: n_c,n_2, k_+k_2, individual fit: k_+k_n.

A) Fitting of PSMα1 kinetic data at 0.25 mg/mL PSMα1 in the presence of 0-3 µg/mL heparin.

B) Fitting of PSMα3 kinetic data at 0.25 mg/mL PSMα3 in the presence of 0-40 µg/mL heparin.

C) Fitting of PSMβ1 kinetic data at 0.025 mg/mL PSMβ1 in the presence of 0-40 µg/mL heparin.

D) Fitting of PSMβ2 kinetic data at 0.25 mg/mL PSMβ2 in the presence of 0.1-1 µg/mL heparin.

E) Fitting of δ-toxin kinetic data at 0.3 mg/mL δ-toxin in the presence of 0.2-0.8 µg/mL heparin fitted to a secondary nucleation dominated model using global constants: n_c,n_2, k_+k_n, individual fit: k_+k_2.

Figure S3

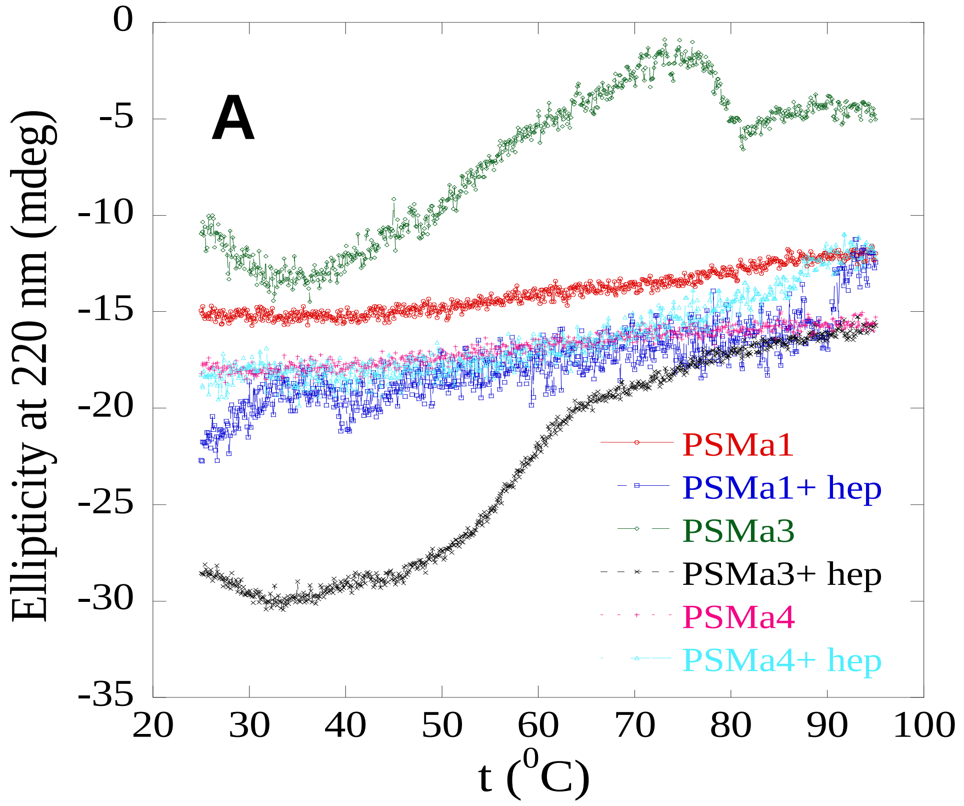

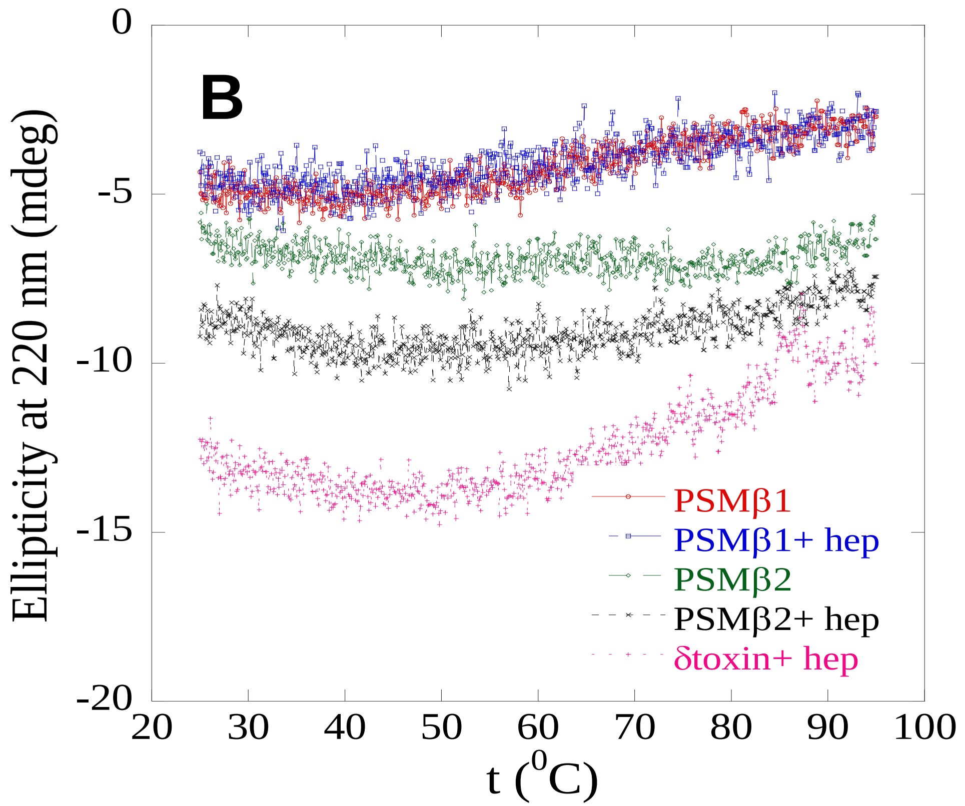

Figure S3: (A) CD thermal scans from 20 to 95°C of α-PSM fibrils incubated in absence and presence of heparin. The concentration of heparin are 3 μg/mL for PSMα1, 40 μg/mL for PSMα3, 50 μg/mL for PSMα4, (B) CD thermal scans from 20 to 95°C of β-PSM and δ-toxin fibrils incubated in absence and presence of heparin (Heparin concentrations: 250 μg/mL for PSMβ1, 1 mg/mL for PSMβ2 and 1 mg/mL for δ-toxin).

Figure S4

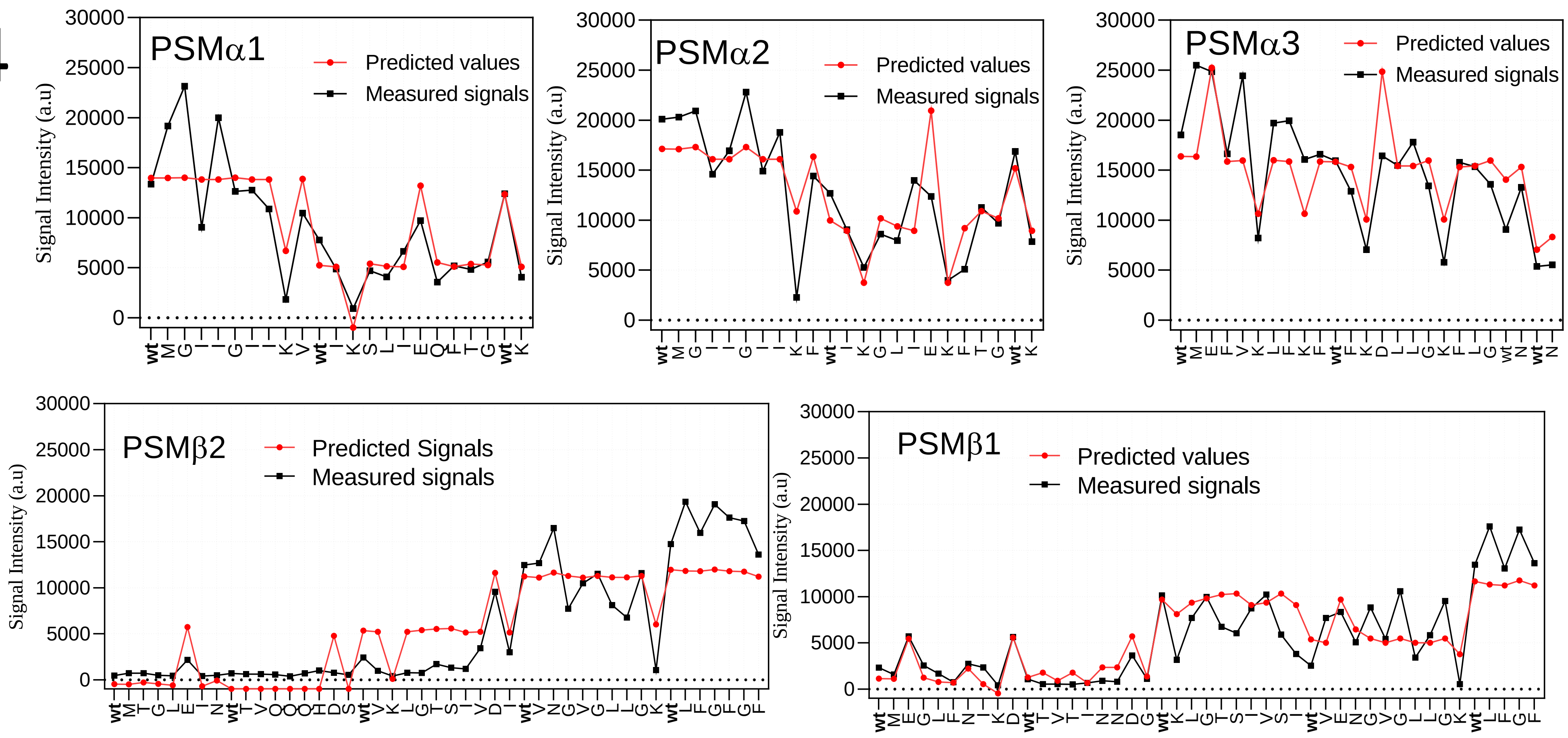

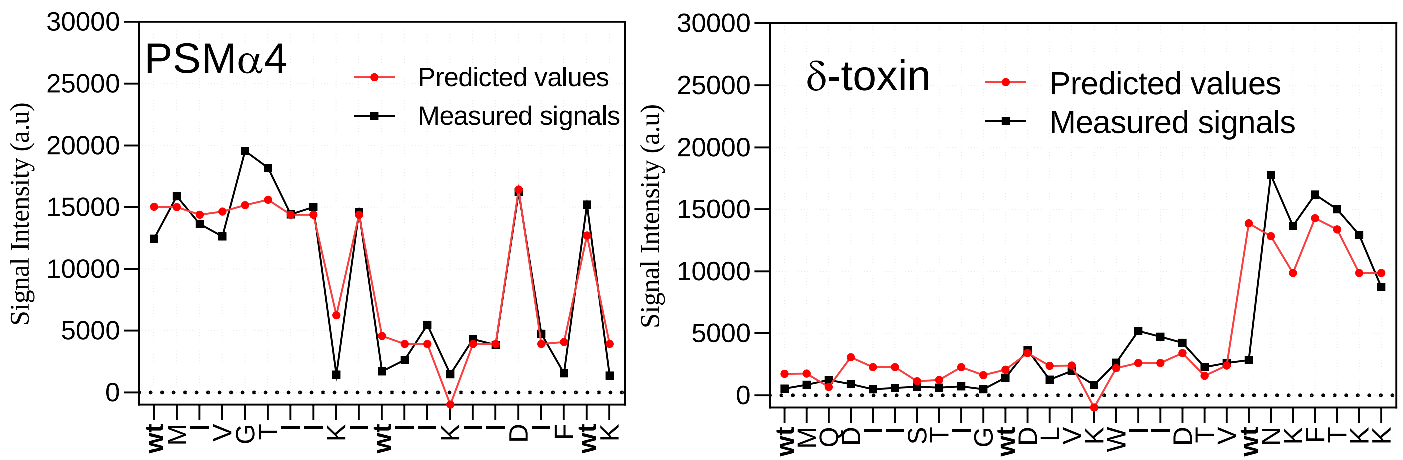

Figure S4. Graphs of predicted and measured signal intensities based on multiple regression with two variables (charge and hydrophobicity) based on data from Ala scan of PSM sequences. Each letter on the x-axis shows the residue that is replaced by Ala.

Supplementary Tables

Table S1. Kinetic parameters of PSM peptide aggregation in the presence of heparin obtained using the webserver Amylofit.

For this analysis, a single global fit was used for all parameters (last row) apart from the compound rate constant k_+k_2 or k_+k_n (as specified in row 2) which is allowed to vary with each heparin concentration (resulting values provided in rows 3-8).

**Heparin (µg/mL)**	**PSMα1**	**Heparin (µg/mL)**	**PSMα3**	**Heparin (µg/mL)**	**PSMβ1**	**Heparin (µg/mL)**	**PSMβ1**	**Heparin (µg/mL)**	**PSMβ2**
	**k_+k_2**		**k_+k_2**		**k_+k_n**		**k_+k_n**		**k_+k_2**
0	6.84×10^{-10}	0	536	0	$3.57\times10^{+17}$	50	$1.52\times10^{+12}$	0	362.00
0.5	1.19×10^{-8}	10	900	5	$2.78\times10^{+17}$	75	$3.66\times10^{+10}$	0.1	20.00
1	1.22×10^{-7}	20	$1.30\times10^{+3}$	10	$2.64\times10^{+17}$	100	$5.04\times10^{+7}$	0.15	10.60
1.5	4.43×10^{-6}	25	$1.75\times10^{+3}$	20	$4.66\times10^{+17}$	150	$4.09\times10^{+3}$	0.2	9.55
2	1.63×10^{-4}	30	$2.64\times10^{+3}$	30	$1.19\times10^{+17}$	200	$1.57\times10^{+5}$	0.25	6.55
3	1.32×10^{-3}	40	$3.75\times10^{+3}$	40	$1.87\times10^{+17}$	250	$1.32\times10^{+4}$	0.5	0.87
m_0 = 110 µM<br>$n_c = 1.98\times10^{-5}$<br>$k_+k_2 = 2.63\times10^{+5}$<br>(conc^{-n2-1} time^{-2})<br>n_2 = 0.542<br>MRE:0.00488		m_0 = 96 µM<br>n_c = 0.723<br>$k_+k_2 = 1.57\times10^{+8}$<br>(conc^{-n2-1} time^{-2})<br>n_2 = 0.695<br>MRE:0.00176		m_0 = 55 µM<br>n_c = 4<br>$k_+k_2 = 2.38\times10^{+4}$<br>(conc^{-n2-1} time^{-2})<br>n_2 = 0.316<br>MRE:0.00418		m_0 = 55 µM<br>n_c = 3.92<br>$k_+k_2 = 6.71\times10^{+5}$<br>(conc^{-n2-1} time^{-2})<br>n_2 = 0.2<br>MRE:0.00905		1	0.53
								m_0 = 5.61 µM<br>n_c = 0.762<br>$k_+k_2 = 3.05\times10^{+4}$<br>(conc^{-n2-1} time^{-2})<br>n_2 = 0.00378<br>MRE: 0.0117	

Table S2. The most important residues for heparin binding identified from Ala scan of PSM peptides

peptide	Mutation[a]	Change in charge[a]	H2-H1[b]
PSMα1	M1A	No change	-0.02
	G2A	No change	0.14
	I4A	No change	-0.76
	K9A	+1 to 0	2.12
	K12A	0 to -1	2.12
	K21A	+1 to 0	2.12
PSMα2	K9A	- (+1 to 0)	2.12
	K12A	- (+1 to 0)	2.12
	K17A	- (+1 to 0)	2.12
	F18A	No change	-0.57
	K21A	- (+2 to +1)	2.12
PSMα3	M1A	No Change	-0.02
	E2A	+1 to +2	1.36
	N21A	No Change	1.4
	K6A	- (+1 to 0)	2.12
	K12A	- (+1 to 0)	2.12
	K17A	- (+1 to 0)	2.12
PSMα4	G5A	No Change	0.14
	T6A	No Change	0.67
	D17A	+ (0 to +1)	1.52
	K9A	- (+1 to 0)	2.12
	K22A	- (+1 to 0)	2.12
δ-toxin	D11A	-1 to 0	1.52
	W15A	No Change	-0.19
	I16A	No Change	-0.76
	I17A	No Change	-0.76
	D18A	-1 to 0	1.52
	N21A	No Change	1.4
	K22A	+2 to +1	2.12
	F23A	No Change	-0.57
	T24A	No Change	0.67
	K25A	+2 to +1	2.12
	K26A	+2 to +1	2.12
PSMβ1	E2A	-1 to 0	1.36
	D10A	-1 to 0	1.52
	D19A	-1 to 0	1.52
	V31A	No Change	-0.46
	E32A	-1 to 0	1.36
	G34A	No Change	0.14
	G36A	No Change	0.14
	G39A	No Change	0.14

	F5A	No Change	-0.57
	K9A	-1 to -2	2.12
	K22A	+1 to 0	2.12
	I30A	No change	-0.76
	K40A	0 to -1	2.12
PSMβ2	E6A	-1 to 0	1.36
	D29A	0 to +1	1.52
	L41A	No Change	-0.44
	G43A	No Change	0.14
	K22A	0 to -1	2.12
	K40A	+1 to 0	2.12

Notes:

[a] Red and green color indicate the mutations led to decrease and increase signal intensity respectively.

[b] H2-H1: Hydrophobicity of 10-mer peptide after alanine mutation – Hydrophobicity of original 10-mer peptide

Table S3. PSM-sequences (used for screening of heparin binding) immobilized on peptide array.

Sequence	Purpose	Sequence	Purpose
MGIIAGIIKV	PSMα1 screen	MEFVAKLFKF	PSMα3 screen
GIIAGIIKVI	PSMα1 screen	EFVAKLFKFF	PSMα3 screen
IIAGIIKVIK	PSMα1 screen	FVAKLFKFFK	PSMα3 screen
IAGIIKVIKS	PSMα1 screen	VAKLFKFFKD	PSMα3 screen
AGIIKVIKSL	PSMα1 screen	AKLFKFFKDL	PSMα3 screen
GIIKVIKSLI	PSMα1 screen	KLFKFFKDLL	PSMα3 screen
IIKVIKSLIE	PSMα1 screen	LFKFFKDLLG	PSMα3 screen
IKVIKSLIEQ	PSMα1 screen	FKFFKDLLGK	PSMα3 screen
KVIKSLIEQF	PSMα1 screen	KFFKDLLGKF	PSMα3 screen
VIKSLIEQFT	PSMα1 screen	FFKDLLGKFL	PSMα3 screen
IKSLIEQFTG	PSMα1 screen	FKDLLGKFLG	PSMα3 screen
KSLIEQFTGK	PSMα1 screen	KDLLGKFLGN	PSMα3 screen
MGIIAGIIKF	PSMα2 screen	DLLGKFLGNN	PSMα3 screen
GIIAGIIKFI	PSMα2 screen	MAIVGTIIKI	PSMα4 screen
IIAGIIKFIK	PSMα2 screen	AIVGTIIKII	PSMα4 screen
IAGIIKFIKG	PSMα2 screen	IVGTIIKIIK	PSMα4 screen
AGIIKFIKGL	PSMα2 screen	VGTIIKIIKA	PSMα4 screen
GIIKFIKGLI	PSMα2 screen	GTIIKIIKAI	PSMα4 screen
IIKFIKGLIE	PSMα2 screen	TIIKIIKAII	PSMα4 screen
IKFIKGLIEK	PSMα2 screen	IIKIIKAIID	PSMα4 screen
KFIKGLIEKF	PSMα2 screen	IKIIKAIIDI	PSMα4 screen
MEGLFNAIKD	PSMβ1 screen	MTGLAEAIAN	PSMβ2 screen
EGLFNAIKDT	PSMβ1 screen	TGLAEAIANT	PSMβ2 screen
GLFNAIKDTV	PSMβ1 screen	GLAEAIANTV	PSMβ2 screen
LFNAIKDTVT	PSMβ1 screen	LAEAIANTVQ	PSMβ2 screen
FNAIKDTVTA	PSMβ1 screen	AEAIANTVQA	PSMβ2 screen

NAIKDTVTAA	PSMβ1 screen	EAIANTVQAA	PSMβ2 screen
AIKDTVTAAI	PSMβ1 screen	AIANTVQAAQ	PSMβ2 screen
IKDTVTAAIN	PSMβ1 screen	IANTVQAAQQ	PSMβ2 screen
KDTVTAAINN	PSMβ1 screen	ANTVQAAQQH	PSMβ2 screen
DTVTAAINND	PSMβ1 screen	NTVQAAQQHD	PSMβ2 screen
TVTAAINNDG	PSMβ1 screen	TVQAAQQHDS	PSMβ2 screen
VTAAINNDGA	PSMβ1 screen	VQAAQQHDSV	PSMβ2 screen
TAAINNDGAK	PSMβ1 screen	QAAQQHDSVK	PSMβ2 screen
AAINNDGAKL	PSMβ1 screen	AAQQHDSVKL	PSMβ2 screen
AINNDGAKLG	PSMβ1 screen	AQQHDSVKLG	PSMβ2 screen
INNDGAKLGT	PSMβ1 screen	QQHDSVKLGT	PSMβ2 screen
NNDGAKLGTS	PSMβ1 screen	QHDSVKLGTS	PSMβ2 screen
NDGAKLGTSI	PSMβ1 screen	HDSVKLGTSI	PSMβ2 screen
DGAKLGTSIV	PSMβ1 screen	DSVKLGTSIV	PSMβ2 screen
GAKLGTSIVS	PSMβ1 screen	SVKLGTSIVD	PSMβ2 screen
MAQDIISTIG	PSMγ screen		
AQDIISTIGD	PSMγ screen		
QDIISTIGDL	PSMγ screen		
DIISTIGDLV	PSMγ screen		
IISTIGDLVK	PSMγ screen		
ISTIGDLVKW	PSMγ screen		
STIGDLVKWI	PSMγ screen		
TIGDLVKWII	PSMγ screen		
IGDLVKWIID	PSMγ screen		
GDLVKWIIDT	PSMγ screen		
DLVKWIIDTV	PSMγ screen		
LVKWIIDTVN	PSMγ screen		
VKWIIDTVNK	PSMγ screen		
KWIIDTVNKF	PSMγ screen		

WIIDTVNKFT	PSMγ screen		
IIDTVNKFTK	PSMγ screen		
IDTVNKFTKK	PSMγ screen		

Table S4. Ala-scanned PSM-sequences immobilized on peptide array

Sequence	Purpose	Sequence	Purpose
MGIIAGIIKV	PSMα1-initial first 10-mer	MEFVAKLFKF	PSMα3 screen
AGIIAGIIKV	PSMα1 -Ala scanning	AEFVAKLFKF	PSMα3 -Ala scanning
MAIIAGIIKV	PSMα1 -Ala scanning	MAFVAKLFKF	PSMα3 -Ala scanning
MGAIAGIIKV	PSMα1 -Ala scanning	MEAVAKLFKF	PSMα3 -Ala scanning
MGIAAGIIKV	PSMα1 -Ala scanning	MEFAAKLFKF	PSMα3 -Ala scanning
MGIIAAIIKV	PSMα1 -Ala scanning	MEFVAALFKF	PSMα3 -Ala scanning
MGIIAGAIKV	PSMα1 -Ala scanning	MEFVAKAFKF	PSMα3 -Ala scanning
MGIIAGIAKV	PSMα1 -Ala scanning	MEFVAKLAKF	PSMα3 -Ala scanning
MGIIAGIIAV	PSMα1 -Ala scanning	MEFVAKLFAF	PSMα3 -Ala scanning
MGIIAGIIKA	PSMα1 -Ala scanning	MEFVAKLFKA	PSMα3 -Ala scanning
IKSLIEQFTG	PSMα1 screen-second 10 mer	FKDLLGKFLG	PSMα3 screen
AKSLIEQFTG	PSMα1 -Ala scanning	AKDLLGKFLG	PSMα3 -Ala scanning
IASLIEQFTG	PSMα1 -Ala scanning	FADLLGKFLG	PSMα3 -Ala scanning
IKALIEQFTG	PSMα1 -Ala scanning	FKALLGKFLG	PSMα3 -Ala scanning
IKSAIEQFTG	PSMα1 -Ala scanning	FKDALGKFLG	PSMα3 -Ala scanning
IKSLAEQFTG	PSMα1 -Ala scanning	FKDLAGKFLG	PSMα3 -Ala scanning
IKSLIAQFTG	PSMα1 -Ala scanning	FKDLLAKFLG	PSMα3 -Ala scanning
IKSLIEAFTG	PSMα1 -Ala scanning	FKDLLGAFLG	PSMα3 -Ala scanning
IKSLIEQATG	PSMα1 -Ala scanning	FKDLLGKALG	PSMα3 -Ala scanning
IKSLIEQFAG	PSMα1 -Ala scanning	FKDLLGKFAG	PSMα3 -Ala scanning
IKSLIEQFTA	PSMα1 -Ala scanning	FKDLLGKFLA	PSMα3 -Ala scanning
MGIIAGIIKF	PSMα2 screen	KDLLGKFLGN	PSMα3 screen
AGIIAGIIKF	PSMα2 -Ala scanning	KDLLGKFLGA	PSMα3 -Ala scanning
MAIIAGIIKF	PSMα2 -Ala scanning	DLLGKFLGNN	PSMα3 screen
MGAIAGIIKF	PSMα2 -Ala scanning	DLLGKFLGNA	PSMα3 -Ala scanning
MGIAAGIIKF	PSMα2 -Ala scanning	MAIVGTIIKI	PSMα4 screen
MGIIAAIIKF	PSMα2 -Ala scanning	AAIVGTIIKI	PSMα4 -Ala scanning

MGIIAGAIKF	PSMα2 -Ala scanning	MAAVGTIIKI	PSMα4 -Ala scanning
MGIIAGIAKF	PSMα2 -Ala scanning	MAIAGTIIKI	PSMα4 -Ala scanning
MGIIAGIIAF	PSMα2 -Ala scanning	MAIVATIIKI	PSMα4 -Ala scanning
MGIIAGIIKA	PSMα2 -Ala scanning	MAIVGAIIKI	PSMα4 -Ala scanning
IKGLIEKFTG	PSMα2 screen	MAIVGTAIKI	PSMα4 -Ala scanning
AKGLIEKFTG	PSMα2 -Ala scanning	MAIVGTIAKI	PSMα4 -Ala scanning
IAGLIEKFTG	PSMα2 -Ala scanning	MAIVGTIIAI	PSMα4 -Ala scanning
IKALIEKFTG	PSMα2 -Ala scanning	MAIVGTIIKA	PSMα4 -Ala scanning
IKGAIEKFTG	PSMα2 -Ala scanning	IIKAIIDIFA	PSMα4 screen
IKGLAEKFTG	PSMα2 -Ala scanning	AIKAIIDIFA	PSMα4 -Ala scanning
IKGLIAKFTG	PSMα2 -Ala scanning	IAKAIIDIFA	PSMα4 -Ala scanning
IKGLIEAFTG	PSMα2 -Ala scanning	IIAAIIDIFA	PSMα4 -Ala scanning
IKGLIEKATG	PSMα2 -Ala scanning	IIKAAIDIFA	PSMα4 -Ala scanning
IKGLIEKFAG	PSMα2 -Ala scanning	IIKAIADIFA	PSMα4 -Ala scanning
IKGLIEKFTA	PSMα2 -Ala scanning	IIKAIIAIFA	PSMα4 -Ala scanning
KGLIEKFTGK	PSMα2 screen	IIKAIIDAFA	PSMα4 -Ala scanning
KGLIEKFTGA	PSMα2 -Ala scanning	IIKAIIDIAA	PSMα4 -Ala scanning
MEGLFNAIKD	PSMβ1 screen	IKAIIDIFAK	PSMα4 screen
AEGLFNAIKD	PSMβ1 -Ala scanning	IKAIIDIFAA	PSMα4 -Ala scanning
MAGLFNAIKD	PSMβ1 -Ala scanning	MTGLAEAIAN	PSMβ2 screen
MEALFNAIKD	PSMβ1 -Ala scanning	ATGLAEAIAN	PSMβ2 -Ala scanning
MEGAFNAIKD	PSMβ1 -Ala scanning	MAGLAEAIAN	PSMβ2 -Ala scanning
MEGLANAIKD	PSMβ1 -Ala scanning	MTALAEAIAN	PSMβ2 -Ala scanning
MEGLFAAIKD	PSMβ1 -Ala scanning	MTGAAEAIAN	PSMβ2 -Ala scanning
MEGLFNAAKD	PSMβ1 -Ala scanning	MTGLAAAIAN	PSMβ2 -Ala scanning
MEGLFNAIAD	PSMβ1 -Ala scanning	MTGLAEAAAN	PSMβ2 -Ala scanning
MEGLFNAIKA	PSMβ1 -Ala scanning	MTGLAEAIAA	PSMβ2 -Ala scanning
TVTAAINNDG	PSMβ1 screen	TVQAAQQHDS	PSMβ2 screen
AVTAAINNDG	PSMβ1 -Ala scanning	AVQAAQQHDS	PSMβ2 -Ala scanning

TATAAINNDG	PSMβ1 -Ala scanning	TAQAAQQHDS	PSMβ2 -Ala scanning
TVAAAINNDG	PSMβ1 -Ala scanning	TVAAAQQHDS	PSMβ2 -Ala scanning
TVTAAANNDG	PSMβ1 -Ala scanning	TVQAAAQHDS	PSMβ2 -Ala scanning
TVTAAIANDG	PSMβ1 -Ala scanning	TVQAAQAHDS	PSMβ2 -Ala scanning
TVTAAINADG	PSMβ1 -Ala scanning	TVQAAQQADS	PSMβ2 -Ala scanning
TVTAAINNAG	PSMβ1 -Ala scanning	TVQAAQQHAS	PSMβ2 -Ala scanning
TVTAAINNDA	PSMβ1 -Ala scanning	TVQAAQQHDA	PSMβ2 -Ala scanning
AKLGTSIVSI	PSMβ1 screen	VKLGTSIVDI	PSMβ2 screen
AALGTSIVSI	PSMβ1 -Ala scanning	AKLGTSIVDI	PSMβ2 -Ala scanning
AKAGTSIVSI	PSMβ1 -Ala scanning	VALGTSIVDI	PSMβ2 -Ala scanning
AKLATSIVSI	PSMβ1 -Ala scanning	VKAGTSIVDI	PSMβ2 -Ala scanning
AKLGASIVSI	PSMβ1 -Ala scanning	VKLATSIVDI	PSMβ2 -Ala scanning
AKLGTAIVSI	PSMβ1 -Ala scanning	VKLGASIVDI	PSMβ2 -Ala scanning
AKLGTSAVSI	PSMβ1 -Ala scanning	VKLGTAIVDI	PSMβ2 -Ala scanning
AKLGTSIASI	PSMβ1 -Ala scanning	VKLGTSAVDI	PSMβ2 -Ala scanning
AKLGTSIVAI	PSMβ1 -Ala scanning	VKLGTSIADI	PSMβ2 -Ala scanning
AKLGTSIVSA	PSMβ1 -Ala scanning	VKLGTSIVAI	PSMβ2 -Ala scanning
VENGVGLLGK	PSMβ1 screen	VKLGTSIVDA	PSMβ2 -Ala scanning
AENGVGLLGK	PSMβ1 -Ala scanning	VANGVGLLGK	PSMβ2 screen
VANGVGLLGK	PSMβ1 -Ala scanning	AANGVGLLGK	PSMβ2 -Ala scanning
VEAGVGLLGK	PSMβ1 -Ala scanning	VAAGVGLLGK	PSMβ2 -Ala scanning
VENAVGLLGK	PSMβ1 -Ala scanning	VANAVGLLGK	PSMβ2 -Ala scanning
VENGAGLLGK	PSMβ1 -Ala scanning	VANGAGLLGK	PSMβ2 -Ala scanning
VENGVALLGK	PSMβ1 -Ala scanning	VANGVALLGK	PSMβ2 -Ala scanning
VENGVGALGK	PSMβ1 -Ala scanning	VANGVGALGK	PSMβ2 -Ala scanning
VENGVGLAGK	PSMβ1 -Ala scanning	VANGVGLAGK	PSMβ2 -Ala scanning
VENGVGLLAK	PSMβ1 -Ala scanning	VANGVGLLAK	PSMβ2 -Ala scanning
VENGVGLLGA	PSMβ1 -Ala scanning	VANGVGLLGA	PSMβ2 -Ala scanning
VGLLGKLFGF	PSMβ1 screen	VGLLGKLFGF	PSMβ2 screen

VGLLGKAFGF	PSMβ1 -Ala scanning	VGLLGKAFGF	PSMβ2 -Ala scanning
VGLLGKLAGF	PSMβ1 -Ala scanning	VGLLGKLAGF	PSMβ2 -Ala scanning
VGLLGKLFAF	PSMβ1 -Ala scanning	VGLLGKLFAF	PSMβ2 -Ala scanning
VGLLGKLFGA	PSMβ1 -Ala scanning	VGLLGKLFGA	PSMβ2 -Ala scanning
MAQDIISTIG	PSMγ screen	DLVAWIIDTV	PSMγ -Ala scanning
AAQDIISTIG	PSMγ -Ala scanning	DLVKAIIDTV	PSMγ -Ala scanning
MAADIISTIG	PSMγ -Ala scanning	DLVKWAIDTV	PSMγ -Ala scanning
MAQAIISTIG	PSMγ -Ala scanning	DLVKWIADTV	PSMγ -Ala scanning
MAQDAISTIG	PSMγ -Ala scanning	DLVKWIIATV	PSMγ -Ala scanning
MAQDIASTIG	PSMγ -Ala scanning	DLVKWIIDAV	PSMγ -Ala scanning
MAQDIIATIG	PSMγ -Ala scanning	DLVKWIIDTA	PSMγ -Ala scanning
MAQDIISAIG	PSMγ -Ala scanning	IDTVNKFTKK	PSMγ screen
MAQDIISTAG	PSMγ -Ala scanning	IDTVAKFTKK	PSMγ -Ala scanning
MAQDIISTIA	PSMγ -Ala scanning	IDTVNAFTKK	PSMγ -Ala scanning
DLVKWIIDTV	PSMγ screen	IDTVNKATKK	PSMγ -Ala scanning
ALVKWIIDTV	PSMγ -Ala scanning	IDTVNKFAKK	PSMγ -Ala scanning
DAVKWIIDTV	PSMγ -Ala scanning	IDTVNKFTAK	PSMγ -Ala scanning
DLAKWIIDTV	PSMγ -Ala scanning	IDTVNKFTKA	PSMγ -Ala scanning
